# A framework for the targeted recruitment of crop-beneficial soil taxa based on network analysis of metagenomics data

**DOI:** 10.1186/s40168-022-01438-1

**Published:** 2023-01-12

**Authors:** Maria Berihu, Tracey S. Somera, Assaf Malik, Shlomit Medina, Edoardo Piombo, Ofir Tal, Matan Cohen, Alon Ginatt, Maya Ofek-Lalzar, Adi Doron-Faigenboim, Mark Mazzola, Shiri Freilich

**Affiliations:** 1 Agricultural Research Organization (ARO), Institute of Plant Sciences, Rishon LeZion/Ramat Yishay, Israel; 2 United States Department of Agriculture-Agricultural Research Service Tree Fruit Research Lab, 1104 N. Western Ave, Wenatchee, WA 98801 USA; 3 University of Haifa, Haifa, Israel; 4 Department of Agricultural, Forest and Food Sciences (DISAFA), University of Torino, Grugliasco, Italy; 5 Department of Forest Mycology and Plant Pathology, Uppsala Biocenter, Swedish University of Agricultural Sciences, P.O. Box 7026, 75007 Uppsala, Sweden; 6 Kinneret Limnological Laboratory (KLL) Israel Oceanographic and Limnological Research (IOLR), P.O. Box 447, 49500 Migdal, Israel; 7 Department of Plant Pathology, Stellenbosch University, Private Bag X1, Matieland, 7600 South Africa

**Keywords:** Microbial community, Metagenomics, Shotgun sequencing, Differential abundance, Microbiome, Rhizosphere, Disease suppressive soils, Rootstock, Biostimulants, Network, Pathway, Compound, Functional annotation, MAG

## Abstract

**Background:**

The design of ecologically sustainable and plant-beneficial soil systems is a key goal in actively manipulating root-associated microbiomes. Community engineering efforts commonly seek to harness the potential of the indigenous microbiome through substrate-mediated recruitment of beneficial members. In most sustainable practices, microbial recruitment mechanisms rely on the application of complex organic mixtures where the resources/metabolites that act as direct stimulants of beneficial groups are not characterized. Outcomes of such indirect amendments are unpredictable regarding engineering the microbiome and achieving a plant-beneficial environment.

**Results:**

This study applied network analysis of metagenomics data to explore amendment-derived transformations in the soil microbiome, which lead to the suppression of pathogens affecting apple root systems. Shotgun metagenomic analysis was conducted with data from ‘sick’ vs ‘healthy/recovered’ rhizosphere soil microbiomes. The data was then converted into community-level metabolic networks. Simulations examined the functional contribution of treatment-associated taxonomic groups and linked them with specific amendment-induced metabolites. This analysis enabled the selection of specific metabolites that were predicted to amplify or diminish the abundance of targeted microbes functional in the healthy soil system. Many of these predictions were corroborated by experimental evidence from the literature. The potential of two of these metabolites (dopamine and vitamin B_12_) to either stimulate or suppress targeted microbial groups was evaluated in a follow-up set of soil microcosm experiments. The results corroborated the stimulant’s potential (but not the suppressor) to act as a modulator of plant beneficial bacteria, paving the way for future development of knowledge-based (rather than trial and error) metabolic-defined amendments. Our pipeline for generating predictions for the selective targeting of microbial groups based on processing assembled and annotated metagenomics data is available at https://github.com/ot483/NetCom2.

**Conclusions:**

This research demonstrates how genomic-based algorithms can be used to formulate testable hypotheses for strategically engineering the rhizosphere microbiome by identifying specific compounds, which may act as selective modulators of microbial communities. Applying this framework to reduce unpredictable elements in amendment-based solutions promotes the development of ecologically-sound methods for re-establishing a functional microbiome in agro and other ecosystems.

Video Abstract

**Supplementary Information:**

The online version contains supplementary material available at 10.1186/s40168-022-01438-1.

## Background

The soil microbiome plays key roles in nutrient cycling through diverse metabolic processes regulating the transformation of organic forms of carbon and nitrogen as well as the availability of essential minerals (e.g., phosphorous) [1–4]. The functioning of the soil microbiome shapes microbial and plant–microbe interactions with ecological outcomes ranging from local (e.g., impacts on soil fatigue and fertility) to global scales (e.g., the emission of greenhouse gases). The sustained plant health observed in native ecosystems is believed to rely, in part, upon a diverse soil microbiome which is supported by the local plant population. This microbial diversity enhances system resilience to disturbances and limits the activity of detrimental biota such as soilborne pathogens. However, the relationship between soil microbiome diversity and ecosystem resilience depends upon the presence of a particular corps of microorganisms possessing specific traits [5–7]. Highly manipulated or managed ecosystems, such as those experienced in crop cultivation, demonstrate reduced productivity over time due to diminished soil fertility, increased pest and disease incidence and negative transformative effects on the soil microbiome leading to disruption of required functions. The rhizosphere soil microbiome is considered a first line of plant protection against the consortia of soilborne microorganisms that can be detrimental to plant health [8]. Indeed, continuous cropping of both annual and perennial plants, in general, leads to a transformation of the soil microbiome that confers increased soilborne pathogen densities and reduced plant productivity [9].

Strategies for surmounting the negative effects of crop monoculture on microbial operations in soil ecosystems have generally relied upon the use of indiscriminate approaches (e.g., soil fumigation) without examination of ecologically sound methods to re-establish a functional microbiome [10]. Attempts to recruit soil microbial resources for use in practices ranging from bioremediation to the control of plant pathogens have largely relied upon inputs with the potential to foster the activity of specific microbial functions. Various soil amendments, in the form of green manure compost and cover crops, are extensively utilized in organic agricultural systems for soilborne disease management [11]. Such soil amendment-based strategies have traditionally been perceived to benefit plant health by providing effective nutritional sources for disease-suppressing microbial populations [12], or via the generation of chemistries that directly suppress pathogenic elements [13]. While several tactics, including vegetation management strategies [14] and the use of organic inputs [15] have been utilized to remediate the composition and function of disturbed soil microbial communities, the complexity of interactions among the diversity of organisms that reside in a soil ecosystem has hindered the ability to successfully direct the trajectory of microbial succession in a manner that leads to the desired function. These limitations are exhibited when instituting methods to establish or engineer a rhizosphere soil microbiome to effectively control plant pathogens in agroecosystems [6, 16].

Strategic management of the soil microbiome has been at the center of study concerning formulation of an ecologically resilient system to control the phenomenon called apple replant disease. The disease is encountered worldwide and not only diminishes the productivity of the current apple orchard but also impedes the successful establishment of new plantings on the site [17]. Apple replant disease (ARD) results from changes in the soil microbiome, including elevation of pathogen populations that are driven by the tree root system with such community transformations occurring rapidly in response to planting apple [18]. The altered microbiome is also characterized by a diminished ability to protect the plant root system from pathogen attack [18]. An intensive research program resulted in establishing protocols for using particular Brassicaceous seed meal (SM) formulations as a soil amendment for effective replant disease management [6, 19, 20]. The SM amendment induces long-term changes in the soil/rhizosphere microbiome that lead to the suppression of specific apple root pathogens [6, 21]. SM formulations had a more long-term persistent effect on the composition of the soil microbiome and corresponding pathogen suppression compared to indiscriminate management approaches such as soil fumigation [6]. The SM-modified microbiome possessed microbial groups with a capacity to metabolize specific compounds, including those present in high concentrations in SM [6]. Despite the apparent advantages of SM-based sustainable disease management approaches, analyses have been absent concerning the functional processes that ultimately yield the composition of the effective microbiome. Systematic explorations of functional changes in the microbiome in response to management practices, such as those induced in soil amendment treatments, can provide essential knowledge for rehabilitating a functional soil microbiome predictably and effectively. The development of more fundamental knowledge concerning metabolite-driven successional trajectories in the soil microbiome leading to pathogen suppression could yield practical means to engineer the indigenous soil microbiome toward enhancing plant health. Further, it will allow for the predictable transfer of approaches to other locations and crop systems [22].

The perception of treatment effectiveness, as dictated by three sides of a triangle, provides a conceptual framework for the study of microorganism activity in soil systems: environmental resources (the dominant resources in a specific amendment treatment), microbial community (species forming possible metabolic conversions repertoire), and function (availability and/or utilization of altered resources). The application of metagenomic sequencing technologies reveals the dynamics of microbial community shifts and enables exploration of their functional outcomes [23]. Metabolic network approaches provide a new framework for translating discrete data from ecological samples into a structured view of biological functions. The subsequent conductance of simulations enables the exploration of associations between the environment and the metabolic potential of the community [24–26].

Similar to genomic approaches, where species-specific metabolic networks are constructed based on the content of enzyme coding genes [27, 28], community networks can be constructed based on the functional annotations of metagenomic data [29]. Network-based simulations allow one to address the influence of changing environmental inputs (e.g., root-specific secreted metabolome, seed meal composition) or the functional repertoire of the community (genomic content in the sample) on the network structure and composition. Iterative simulations can be applied for delineating functional divisions between community members, such as co-dependencies on utilization or generation of specific metabolites and hierarchical cross-feeding interactions [30–34]. For example, in a previous study, we applied a network approach for analyzing metagenomic data from rhizosphere soil *vs* bulk soil, the latter not under the direct influence of plant roots [29]. Root-specific effects linked the utilization of distinct root exudates (e.g., flavonoids, organic acids) with particular taxonomic groups. Many of these associations are well supported in the literature, evidencing the impact of such compounds on microbial community structure. Such simulations of community-level metabolic activity enable the prediction of niche modifications that encourage a desired function (e.g., the potential to suppress disease progression) [22].

**Fig. 1:**
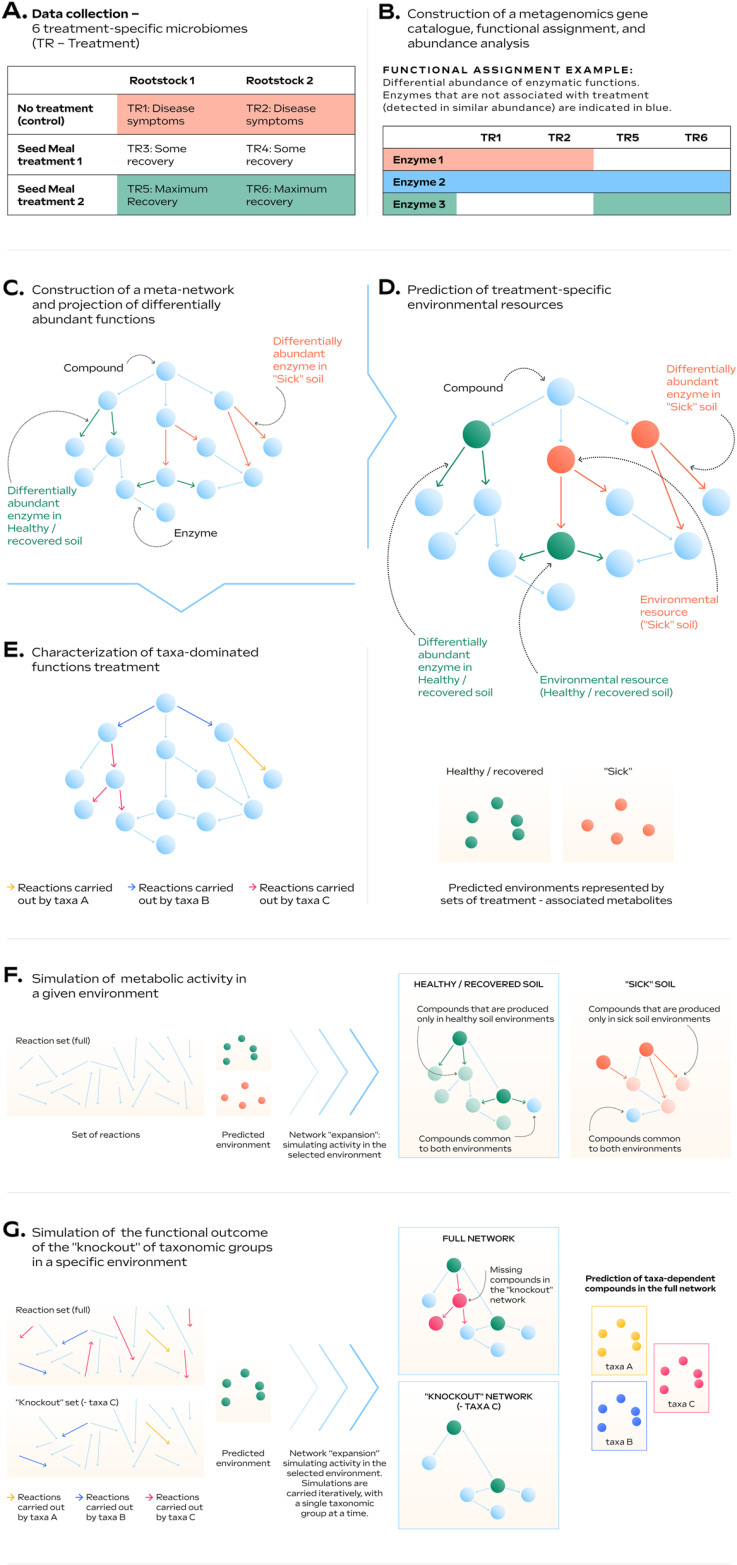
Workflow of the data analysis. Full details of the process described in panel (**D**) (prediction of treatment specific environmental resources) is detailed in Additional file 2: Figure S2

In this study, we applied a network-based metagenomic analysis of community metabolism to understand the processes shaping the causal microbiology of apple replant disease and the metabolic mechanisms supplied or induced by the SM amendment, which function to yield successful disease suppression. Here, we provide an example of the workflow that is used in this analysis (Fig. 1). Reproducibility of effective disease control in response to the application of biologically-based non-fumigant approaches has been a consistent limitation to adopting sustainable strategies in commercial agricultural systems. Although Brassicaceous SM amendments have repeatedly shown replant disease control across diverse orchard systems employing various apple rootstock genotypes [5, 6, 19, 20], optimal disease suppression is attained when an appropriate rootstock is employed [35]. Plant genotype-specific differences in rhizosphere-related traits, including metabolic profiles of root exudates [36], may have a positive or negative effect on the composition and function of the microbiome [37]. We hypothesize that the success of a soil amendment treatment is determined by the direct-supply and induced-generation of metabolites that are beneficial to functional-organisms contributing to disease control or deleterious to organisms contributing to disease progression.

## Methods

### Experimental design: SM treatments, phenotyping, and preparation of libraries

Experimental design is as described in Somera et al., 2021 [38]: briefly, experiments were conducted in a greenhouse using replant orchard soil collected on 11 October 2017 from the GC commercial orchard located near Manson, Washington, USA (latitude 47° 53′ 05″ N, longitude 120° 09′ 30″ W). In this experiment, we evaluated the effects of Brassicaceous SM amendments, including *Brassica napus* (canola) and a 1:1 formulation of *Brassica juncea* (brown mustard) and *Sinapis alba* (white mustard) (BjSa SM) applied at a rate of 4.4 t ha^−1^, on the apple rootstock rhizosphere soil metagenome. The experiment included a no-treatment control (NTC). Soils were planted with a susceptible (M26) and tolerant (G210) apple rootstock with five replicates for each soil treatment/rootstock combination (Table 1). Pots were arranged in a complete randomized block design with 10 pots per block (5 replicates × 2 genotypes). At harvest, 4 months post-planting, a rhizosphere soil sample was collected from each rootstock. Plants were removed from pots and shaken to remove soil that was loosely adhering to root surfaces. Soil firmly attached to the roots was collected from multiple locations along the root system using sterile tweezers and a scoopula. DNA was extracted according to the manufacturer’s instructions from 0.25 g of rhizosphere soil per plant using the DNeasy PowerSoil Kit (Qiagen). The metagenomes analyzed in this study were created using the same DNA samples as in Somera et al. 2021 [38]. Paired-end libraries were prepared by the sequencing facility using the Illumina DNA Prep workflow (formerly named Nextera DNA Flex) with 50 ng DNA as input. The experiment consisted of five replicates for each rootstock genotype/soil treatment combination with a total of 30 DNA samples.

**Table 1:** Summary of variations in plant health and microbiome composition for the experimental samples used in this study as described in [38]

Treatment/root stock	M26	G210
No treatment control (NTC)	Sick (no disease suppression)^ ±^	Sick (no disease suppression) ^±^
*Brassica napus* SM	Partial disease suppression ^±^ /shift in community structure*	Partial disease suppression ^±^ /shift in community structure*
*Brassica juncea/Sinapis alba* (BjSa) SM	Maximal disease suppression ^±^ /shift in community structure*	Maximal disease suppression ^±^ /shift in community structure*

^±^Assessment of disease suppression was based on plant growth performance as well as the occurrence of causal agents in root tissue

^*^Relative to control treatment

### Metagenomic data processing: sequencing, assembly, annotation, differential abundance analysis, and identifying functions that are dominated by specific taxonomic groups

Shotgun metagenomic sequencing was conducted at the University of Illinois-Roy J. Carver Biotechnology Center using the Illumina NovaSeq 6000 sequencing system. 11.5 billion quality reads at a length of 150 bp were sequenced (an average of ~ 383 million reads per sample; Additional file 1). Raw reads were filtered and cleaned using Trimmomatic [39] for removing adapters and the FASTX-Toolkit (version 0.0.13.2) for (1) trimming read-end nucleotides with quality scores < 30 using fastq_quality_trimmer and (2) removing reads with < 70% base pairs with quality score ≤ 30 using fastq_quality_filter. Following filtration, clean reads were assembled using MEGAHIT program (version v1.1.3) [40]. Each of the six treatments was assembled independently (Additional file 2: Table S1). Prodigal software (version 2.6.2) [41] was used for detection of ORFs (Additional file 2: Table S2). To assign taxonomic and functional annotations, all detected ORFs were searched against the non-redundant NCBI protein database using the diamond algorithm v0.9.24 [42]. Results were then uploaded to MEGAN software (version 6.13.4) [43]. The LCA algorithm was applied (parameters used with a minimum bit-score of 150, minimum support of 1% top percent threshold, and maximal expected 1 × 10^−5^) to compute the assignment of CDSs to specific taxa and to the KEGG [44] functional scheme including assignment of KO and EC accessions for enzyme coding genes (Additional file 2: Table S2). Contig-level taxonomic assignments were inferred from the gene-level annotations. In 94% and 97% of the contigs, all genes were consistent in their taxonomic assignments in the genus and order level, respectively (Additional file 2: Table S3). Ambiguities were resolved by assigning the contig with the most frequent annotation of its associated genes. For each assembly (experiment), a count table was constructed by mapping all five libraries in each assembly to the contigs using BWA mem [45] apping software (v0.7.17) with default parameters. Based on the MEGAN annotations, a unique conversion count table was constructed for each annotation scheme used by binning contigs according to the annotation key used (taxonomic and various functional schemes such as EC and KOs). Next, all treatments were merged into joint count tables, based on either functional or taxonomic keys. Next, for each annotation key, count tables from each experiment were merged into a single count table for all 30 samples sequenced (6 treatments × 5 replicates). Significance of differential abundance (DA) of the reads associated with the respective contigs (e.g., all contigs assigned to a specific genus) in each experiment across the respective replicates was determined using edgeR function implemented in R, requiring FDR adapted *P* value < 0.05. DA taxonomic groups are listed in Additional file 3; DA functions are listed in Additional file 4.

To associate enzymatic functions (accessions associated with EC assignments) with specific microbial groups, we retrieved for each EC accession the taxonomic annotations of all its associated reads. The Simpson index [46], typically used to determine species dominance in ecological surveys, was applied here to determine the dominance of specific taxonomic groups in regard to a function, as described in [29]. To this end, instead of looking at the frequencies of species in a sample (as in ecological surveys), for each enzyme (equivalent to a sample), we looked at the distribution of the taxonomic affiliations of its associated reads (equivalent to a species). Simpson Indices were calculated for each enzyme in the dataset for each of the original samples. Then, a treatment-specific Simpson index value was determined for each enzyme by calculating mean values across corresponding samples. Finally, within each treatment, we described a function to be dominated by a taxonomic group if (i) the mean Simpson index across all samples value was greater than 0.4; and (ii) the same taxonomic group was dominant in all replicate samples. Hence, associations between taxonomy and function are representative of a treatment and are consistent between replicates. Lists of taxa-dominated enzymes at the order and genus levels from the G210 rootstock across three treatments (NTC, BjSa and *B. napus*) are provided in Additional file 5. When the sets of taxa-dominated enzymes were correlated with the corresponding sets of differentially abundant enzymes from the same treatment (e.g., taxa-dominated enzymes in BjSa SM correlated with DA enzymes in BjSa SM), there was a significant overlap between sets (Additional file 2: Figure S1). In comparison, reciprocal crosses (e.g., taxa-dominated enzymes in BjSa SM correlated with DA enzymes in NTC samples) did not show such overlaps, suggesting that taxa-dominated enzymes have a functional impact in the corresponding samples. Network distribution of the taxa-dominated enzymes exhibited similar topological patterns to common, non-dominated functions and taxonomic diversity scores did not significantly correlate with enzymes’ connectivity (number of neighbors) (Additional file 6).

A detailed reproducible bioinformatics workflow for processing metagenomics data, differential abundance analysis, and identification of functions that are dominated by specific taxonomic groups is available at https://github.com/ot483/NetCom2 (steps 1–8).

### Network construction, prediction of treatment-specific environmental resources, and simulation of metabolic activity

A meta-network was constructed, containing all enzymatic functions annotated across all samples following the procedure outlined in [27–29]. The network was constructed by mapping enzymes to metabolic reactions based on a scheme downloaded from the KEGG database [44] in June 2016. Directional edges represent reactions connected by common metabolites (nodes, Fig. 1C). The set of metabolic reactions and its organization in the metabolic network it forms reflect nutritional dependencies on the environment [47]. Topological analysis of the metabolic networks with the graph theory-based strongly connected components (SCC) algorithm was applied to predict the set of environmental resources (the dominant resources/metabolites in a specific treatment acquired from the environment) [48]. An environmental proxy was generated for three networks: the full meta-network and two sub-networks of differentially abundant reactions. The environmental proxy is a list of metabolites that are predicted to be externally consumed from the environment (‘environmental resources’, Fig. 1D). Predictions are based on the implantation of Tarjan’s SCC [25] through its implantation in NetCom [28]. Since the treatment-specific sub-networks were constructed based on differentially abundant enzymes only, they are highly fragmented, leading to a prediction of artificial source-metabolites [29]. Hence, metabolites representing environmental resources that were identified for treatment-specific sub-networks were compared to those identified for the full meta-network (Additional file 2: Figure S2). Only metabolites present in both sets were further considered within the environment proxy list.

These environmental resource lists were then used to further explore the metabolic activities in each treatment by applying the Expansion algorithm [27, 49]. Briefly, the algorithm can be used to predict feasible reactions in a metabolic network (expanded) given a pre-defined set of substrates and reactions. The algorithm starts with a set of source-metabolites acting as substrates (i.e., the environmental resource list); it scans the reaction bank for feasible enzymatic reactions for which all the possible substrates exist; all feasible reactions are added to the network, their products being the substrates for the next set of reactions. The network stops expanding when no feasible reactions are found. Thus, the full expansion of the network reflects both the reaction repertoire and the primary set of compounds (predicted source metabolites). Here, simulations of metabolic activity were carried out by expanding the full set of reactions detected across all samples (meta-network) while using treatment-specific sets of environmental resources (source metabolites). That is, expansion iterations were carried out using sets of predicted environmental resources representing the treated samples vs control samples (Fig. 1F). The treatment-specific expanded networks are provided in Additional file 7.

The expanded network from the BjSa X G210 treatment (Additional file 7) was used as a reference for community ‘knock outs’ simulations in which selected taxonomic groups were removed (Fig. 1G). In each of the removal iterations, all edges (enzymes representing metabolic functions) specifically dominated by a taxonomic group (i.e., taxa-dominated enzymes) were removed from the original enzyme set. The impact of the removal of each such group was estimated according to differences in the metabolite content (number of metabolites) between the network expanded from the truncated enzyme set, and the reference meta-network. The removed metabolite vectors (Additional file 8), created for each iteration, were mapped to KEGG pathways. Enrichment of treatment-specific networks in components (nodes, edges) associated with particular pathways was determined using the enricher function in R requiring a *p* value <  = 0.05. *P* values were adjusted for multiple comparisons using the “fdr” adjustment method available in the *P*.adjust function in R.

A detailed reproducible bioinformatics workflow for network construction, prediction of treatment-specific environmental resources, simulation of metabolic activity, and network visualization is available at https://github.com/ot483/NetCom2 (steps 9–11).

### Visualizations

Principal coordinate analysis (PCoA) was performed using R prcomp function in R stats package, version 3.6.3. Data for PCoA were analyzed within the R environment (R Version 4.0.3 in RStudio Version 1.4.1103). Count tables were filtered to at least 50 counts per feature, and rarefied (with vegan 2.5–7 package), to correct for differences in sampling depth (library size) between samples. PCoA plots were based on Bray–Curtis dissimilarity (beta diversity) and were computed using labdsv 2.0–1 package. PCoA plots were made using the ggplot2 package version 3.3.2. Meandist function in vegan was used to summarize the Bray–Curtis distance matrix, and create tree plots of the treatments (Additional file 2: Figure S3). Table 2 was created using package gt version 0.2.1. All network visualizations were made using Python 3.6 NetworkX 2.5 and Matplotlib 3.3.2 packages.

**Table 2:**
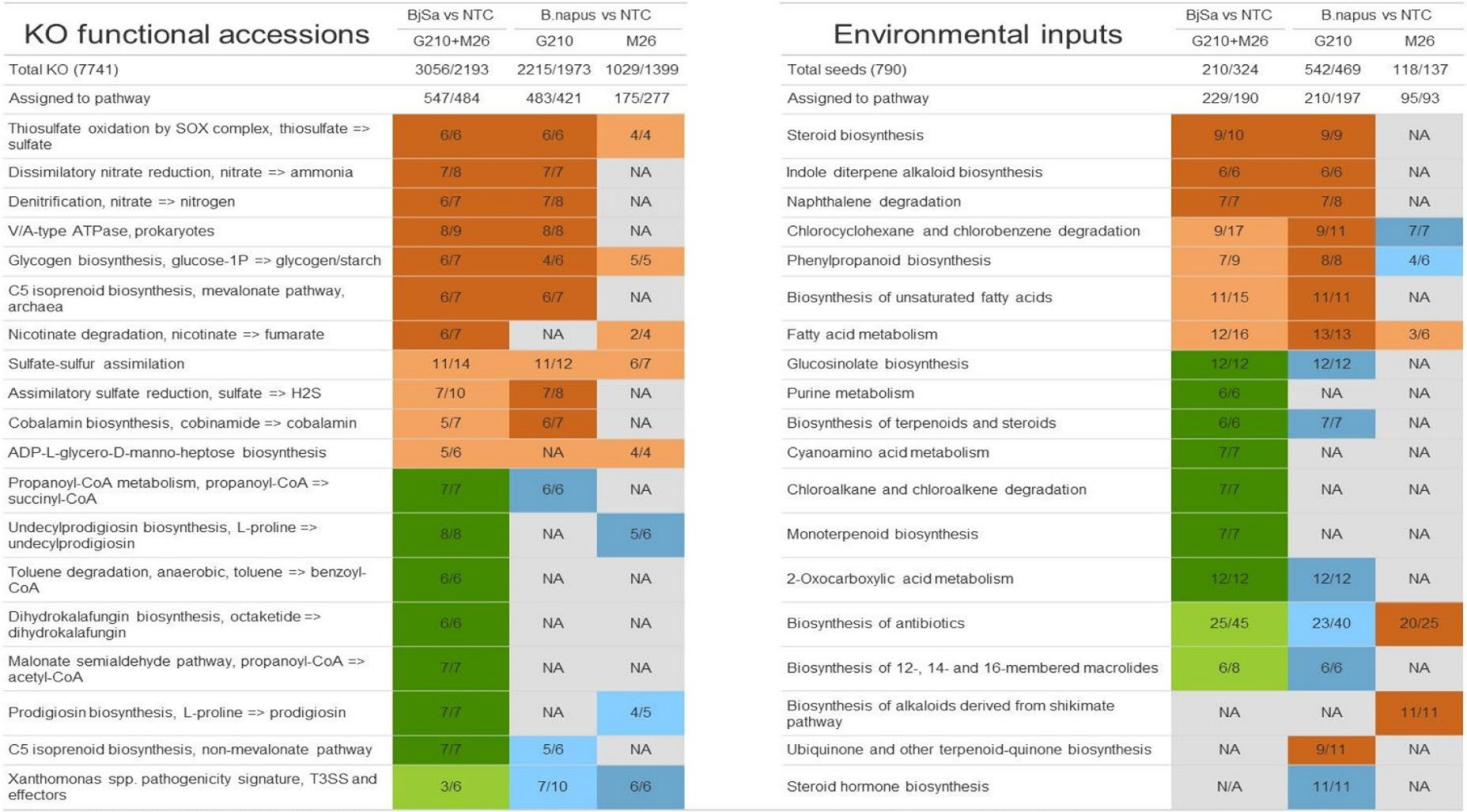
Biological processes significantly enriched with treatment versus control entities

Left: KEGG modules enriched with differentially abundant KO accessions

Right: KEGG pathways significantly enriched with treatment-specific environmental resources. Processes that are enriched in NTC (control) entities are colored in orange; processes that are enriched in Brassica juncea/Sinapis alba (BjSa) seed meal (SM) treatment entities are colored in green; processes that are enriched in *Brassica napus* SM treatment entities are colored in blue. Dark coloring indicates significance (FDR adjusted *P* value <  = 0.05); bright coloring indicates the experimental conditions with which the majority of entities are associated (thought below significance threshold). Only pathways with at least six entities with clear dominance (≥ 85%) of one of the treatments and exhibiting significance in at least one of the comparisons tested are shown. NA denotes pathways with less than six entities assigned

### Evaluation of specific metabolites as biostimulants/biosuppressors of beneficial taxonomic groups

Metabolite enrichment experiments were carried out using the same SM amendment protocol and the same orchard soil as in the original experiment [38]. The compounds tested were dopamine and vitamin B12. Soil treatments included BjSa SM, BjSa SM + compound, NTC, and NTC + compound. For soil enrichment experiments with dopamine, the compound was applied to the soil 1 week after SM amendment (5 weeks prior to planting). In the vitamin B12 experiment, however, the soil was conditioned closer to the time of planting (5 weeks post-SM amendment and 1 week prior to planting). This adjustment was made in order to try to minimize depletion of the compound over time in the bulk soil and maximize shifts to the rhizosphere microbiome. For soil enrichment with dopamine, a final concentration of 30 µg ml^−1^ was obtained by dissolving 0.075 g of dopamine hydrochloride (Sigma-Aldrich, Inc., St. Louis, MO, USA) into 50 ml of sterile DI water. The entire 50 ml volume was then applied to 2.5 L of soil using a chromatography sprayer while continuously mixing by hand. The concentration of dopamine applied to soil was largely based on studies in which catecholamines added to liquid growth media stimulated or promoted the growth of bacterial cultures [50, 51]. Similarly, the amount of vitamin B12 applied to soil was based on concentrations of vitamin B12 produced by Rhizobiales isolates when grown in pure culture [52, 53]. For soil enrichment with vitamin B12, a final concentration of 10 mg per gram of soil was obtained by dissolving 25 mg of vitamin B12 (Cyanocobalamin; Sigma-Aldrich, Inc., St. Louis, MO, USA) into 50 ml of sterile DI water. The entire 50 ml volume was then applied to 2.5 L of soil as described above. Each soil treatment was represented by 10 replicates.

Treated soils were dispensed into large plastic containers (cell diameter of 6.35 cm and a depth of 25.4 cm) and placed in a standing rack in a randomized complete block design. The soils were then placed in growth chambers for a period of weeks with temperature maintained at 18–22 °C. Apple seed germination and preparation of Gala apple seedlings were conducted as previously described [54]. A single 8-week-old apple seedling was planted into each individual soil cell at 6 weeks post-SM treatment (5 weeks post-dopamine application/1 week post-vitamin B12 application). This time frame was necessary to allow for degradation/aeration of herbicidal chemistries that are generated in response to seed meal incorporation into moist soil. Upon harvest, growth measurements of apple seedlings were made for root biomass, shoot biomass, and total plant biomass (fresh weight).

Rhizosphere soil samples were collected as described above at 4 weeks post-planting and at harvest (8 weeks post-planting). DNA was immediately extracted from these soil samples (0.25 g) using the Qiagen DNEasy PowerSoil Kit. Bacterial amplicon libraries were generated and analyzed as described in Somera et al. 2021 [38]. In summary, the DNA extracted from rhizosphere soil was sent directly to the sequencing facility (Molecular Research, Shallowater TX, USA). DNA was PCR-amplified prior to library preparation. Bacterial 16S rRNA regions were sequenced on an Ion Torrent S5 XL (20,000 reads per sample) using the bacterial tag-encoded FLX amplicon pyrosequencing (bTEFAP®) method (Dowd et al. 2008). Following removal of barcodes, primers, sequences < 150 bp, those containing ambiguous base calls and those with homopolymer runs exceeding 6 bp, sequences were quality filtered using a maximum expected error threshold of 1.0, dereplicated and denoised. This was followed by the removal of chimeric reads. Processed sequences were clustered into OTUs at the 97% similarity level. As part of the sequencing service, the taxonomic classification of OTUs was performed using a curated database derived from RDP-II (https://rdp.cme.msu.edu/) and NCBI (https://www.ncbi.nlm.nih.gov). Analysis of 16SrRNA amplicon sequence data was performed as described in Somera et al. 2021 [38]. In short, microbial community profiles as influenced by soil treatment were evaluated using Explicet software package version 2.10.5 [55]. Before the OTU read counts analysis, data were edited to remove singletons and doubletons as well as all eukaryotic and archaeal OTUs.

In order to estimate the absolute abundance of taxonomic groups, 0.25 g of rhizosphere soil was weighed out for each sample (*n* = 5 per treatment) and DNA was extracted using the DNeasy PowerSoilPro Kit (Qiagen) according to the manufacturer’s instructions. The concentration of DNA extracted (ng/ul) was normally distributed within each experimental treatment according to Shapiro–Wilk and Kolmogorov–Smirnov normality tests, so ordinary one-way ANOVA was used to test for significant differences among treatment means. No significant differences in DNA concentration were identified between any of the 4 treatments (*p* = 0.38). Therefore, we assumed DNA extraction efficiency was similar between treatments. The primer pair 799F/1193R was then used to amplify the bacterial 16S rRNA region [56]. Quantification of total bacteria was conducted using the StepOnePlus Real-Time PCR System, with the following run conditions: 10 min at 95 °C followed by (30 s at 95 °C, 1 min at 58 °C) × 40 cycles. PCR reactions contained 1 µL DNA extract diluted 1:100, 3 µL SYBR Green PCR Master Mix (Applied Biosystems, Warrington, UK), 0.05 µL of each primer [100 µM] and 5.9 µL nanopure water. Purified genomic DNA from *Pseudomonas florescence* (isolate # SS101) was used to generate the standard curve with a dilution range from 0.01 to 100 pg µL^−1^. Each set of qPCR reactions included a no template control; all reactions were performed in triplicate. Absolute values of total bacterial DNA (16S rRNA gene DNA) obtained via quantitative PCR (qPCR) were used to transform the relative abundances of selected bacterial genera within the Orders Xanthomonadales and Nevskiales to absolute values. Significant differences in the absolute abundance of 16S rRNA gene DNA were identified between treatments using the Kruskal–Wallis test followed by Dunn’s multiple comparisons test.

## Results and discussion

### Characterization of ‘sick’ vs ‘healthy/recovered’ microbiomes

**Fig. 2:**
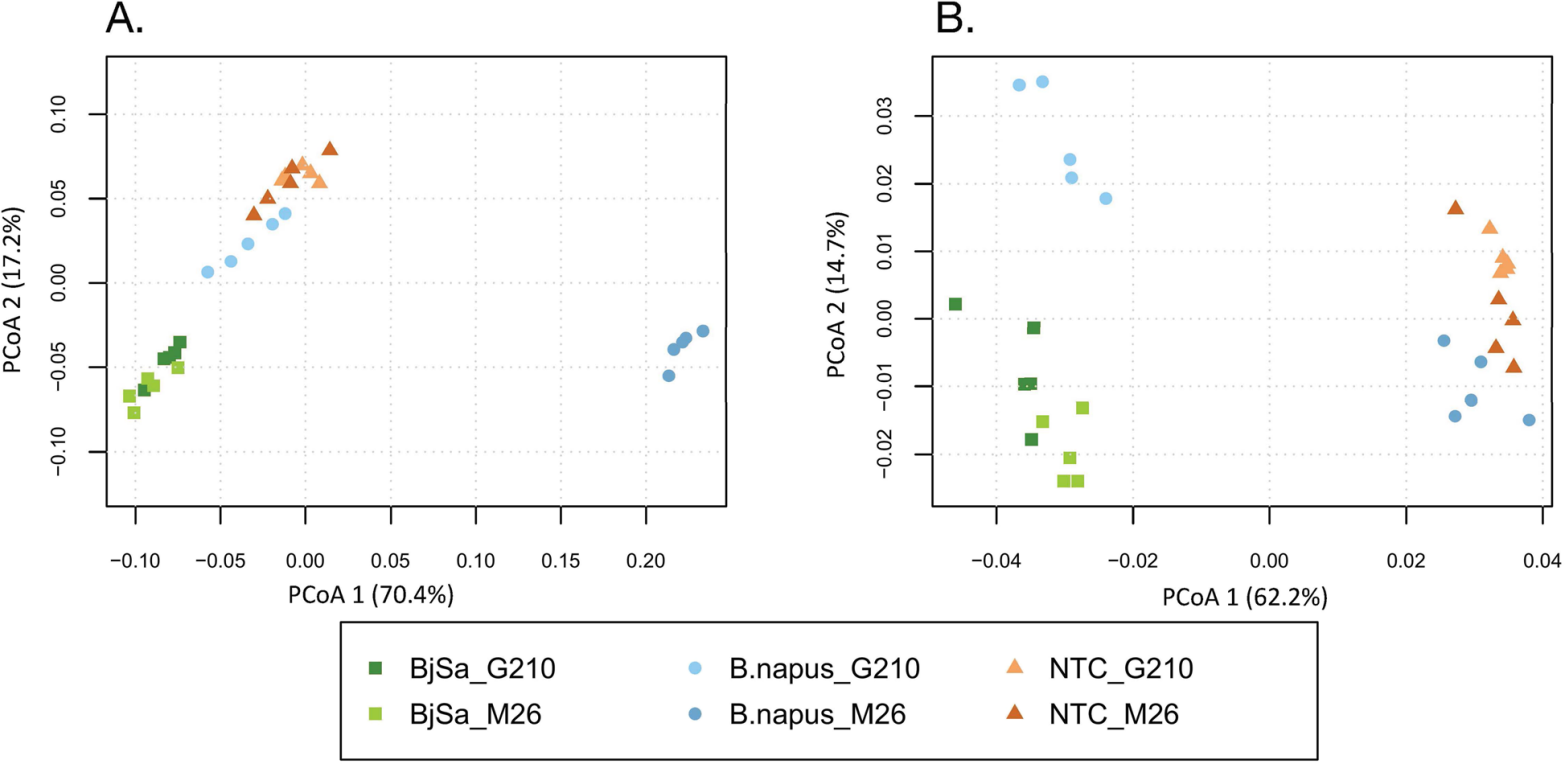
Principal coordinates analysis (PCoA) plots of Bray–Curtis dissimilarities in the taxonomic (**A**) and functional (**B**) groups in root bacterial communities based on count tables derived from the metagenome analysis. **A** Genus level taxonomy—overall 2630 genera were detected across all 30 samples; similar pattern was observed when using order level as a key for merging the table (Additional file 2: Figure S5). **B** Functional annotations. Several schemes were used for functional annotation including NOG and SEED and KEGG EC (Additional file 2: Figure S5). Here, we show ordination pattern based on KO annotations. Overall 7741 unique KO categories were identified across all 30 samples. Percentage of variance are indicated in brackets. The treatments were additives (*Brassica juncea/Sinapis alba* seed meal (BjSa), *Brassica napus* seed meal and non-treated control-NTC), with two apple rootstocks (M26, G210). Both databases were filtered to at least 50 counts per feature and rarefied

The current research was designed as a comparative study of rhizobiome communities from ‘sick’ vs ‘healthy/recovered’ apple rootstocks. Rhizobiome communities obtained from rootstocks grown in orchard soil with a documented history of replant disease were termed ‘sick’ while those obtained from plants grown in replant soil amended with established SM treatments known to suppress the disease were termed ‘healthy/recovered’. Susceptible and tolerant rootstocks (M26 and G210, respectively) were grown in orchard soil amended with either *Brassica juncea*/*Sinapis alba* (BjSa) or *Brassica napus* seed meal. BjSa and *B. napus* SM are known to induce highly effective and less effective/variable levels of replant disease control, respectively. Overall, the data includes the six treatments summarized in Table 1. For each treatment × rootstock combination, disease suppression and growth performance were assessed at the end of the study. Microbial community structure and function were previously assessed based upon bacterial 16S rRNA and fungal/oomycete ITS sequences as described in [38]. In general, BjSa SM was superior to *B. napus* SM in terms of suppressing soilborne pathogens. Both SM treatments significantly affected the taxonomic composition of the soil microbiome [38]. Here, we report the analysis of deep shotgun metagenomic sequencing from the same rhizosphere soil samples. A total of 1.7 Tbp were sequenced from 30 samples, including 6 treatments with 5 replicates each. Each of the six treatments was assembled independently. Between 61 and 73% of the reads were mapped to 9717277 contigs longer than 2 Kbp with N50 and average contig length ranging from 4742 to 5272 bp and 4458 to 4756 bp, respectively (Additional file 2: Table S1). The mapping of a significant part of the data to long fragments, longer than the average bacterial gene, supported gene identification and conductance of gene-centric functional analysis. Overall the total number of basepairs sequenced, the number of contigs and their average length pose this assembly as one of the most comprehensive soil metagenomes currently available, considering relevant depositories such as https://webapp.ufz.de/tmdb/ [57]. Approximately eight million metagenes (per assembly) were predicted from this set of long contigs (Additional file 2: Table S1). Taxonomic annotations were assigned to 57% of the genes (Additional file 2: Table S2). Estimates of community composition made from amplicon [38] and metagenomic sequences (Additional file 9) were highly concordant at the phylum level (Additional file 2: Figure S4). Beyond the similarity in the relative abundance of key phyla, deep sequencing led to approximately a five-fold increase in the number of groups identified at the phyla level compared to amplicon sequencing and approximately a three-fold increase at the genus level (Additional file 2: Table S4). Multidimensional scaling (PCoA analyses) of metagenomic abundance data at the genus (Fig. 2A) and order (Additional file 2: Figure S5) levels confirmed that both SM amendments induced a shift in microbial community structure and showed co-clustering patterns that concurred with the respective phenotypic classification (severity of disease symptoms, Table 1). In the BjSa SM and NTC samples, clustering patterns reflected a stronger amendment effect than rootstock effect, in which all replicates from the same treatment (and different rootstocks) were co-clustered. In the *B. napus* SM treatment, however, there was a strong rootstock effect on the taxonomic composition of the rhizobiome.

Next, we screened for microbial groups that were significantly enriched or depleted under different experimental conditions. Considering the high diversity of the soil community, the introduction of chimeric contigs constructed from different strains of the same species is common in metagenomics assemblies [58]. To avoid false classifications associated with high resolution annotations, we conducted an analysis at the genus level and higher. Overall, 669 and 434 genera were identified as being enriched in BjSa or *B. napus* SM treatments relative to NTC, respectively (Additional file 3). Most genera enriched by SM were representatives of the phylum Actinobacteria (Additional file 2: Figure S6), which concurs with previous reports on significant differences in abundance of microbial taxa associated with replant vs non-replant soil [59] and SM treated vs. non-treated replant soil [6, 60]. Treatment-enriched genera matched many of the groups also detected by the 16S rRNA survey of the same samples [38]. Many of these groups have documented activity against ARD pathogens. Both SM-treated samples were enriched with sulfide-oxidizing genera (e.g., *Thiomicrospira*) whereas NTC samples were enriched in many sulfur-reducing groups including *Desulfobacteracea* and *Desulfobulbus*. Taxa that were highly abundant in SM-treated samples also included reported plant growth-promoting bacteria such as *Variovorax, Azospirillum, Klebsiella, Enterobacter*,* Alcaligenes*,* Arthrobacter*,* Burkholderia*, and *Bacillus* as well as nitrogen-fixing bacteria including *Frankia, Bradyrhizobium, Mesorhizobium*, and *Sinorhizobium* [61–63]. Finally, according to our amplicon data, the fungal groups *Arthrobotrys* (nematode-trapping fungus) and *Trichoderma* were highly abundant in the SM-treated samples. Notably, *Brassica* SM treatments are not equally suppressive to all pathogens. In this analysis, and consistent with the results from amplicon sequencing, high levels of *Pythium,* previously reported to be stimulated by *B. napus* [20, 38, 60], were detected in the *B. napus* but not BjSa SM treatment, relative to non-amended controls. Also, the amplicon data shows that arbuscular mycorrhizal fungi (AMF) of the Phylum Glomeromycota were significantly more abundant in SM amended soil, with members of the order *Paraglomerales* enriched in BjSa but not in *B. napus* SM treatments relative to the NTC (Additional file 3).

### Associating specific functions with ‘sick’ vs ‘healthy/recovered’ microbiome

Microbiome-mediated plant recovery is thought to rely on the increased abundance of species with particular functional characteristics [6, 38]. Beyond confirming the identity of previously reported taxonomic groups as differentially abundant in SM-induced microbiomes [5, 6, 38], we aimed to delineate the unique functional contributions of these groups leading to recovery from replant disease. Out of ~ 48,000,000 genes, 39 to 82% were assigned to a functional category, considering different annotation schemes (Additional file 2: Table S2). The functional profile was consistent with the taxonomic profile (Fig. 2B), pointing to seed meal-induced shifts in microbiome performances. As in Fig. 2A, the rootstock effect on microbial community function is most observable for the *B. napus* SM treatment in comparison to BjSa SM and NTC treatments.

In order to characterize key functional differences between ‘sick’ and ‘recovered’ samples, we first focused on BjSa SM and NTC treatments, with the weaker rootstock effect, and identified KEGG modules that were enriched in differentially abundant KO accessions (Fig. 2B, Additional file 4). In parallel, in the *B. napus* treated samples, each rootstock was compared to the respective NTC samples. Functional modules enriched in differentially abundant (DA) KOs are listed in Table 2. In accordance with Fig. 2B, a highly similar set of KO modules was enriched in NTC treatments relative to BjSa and *B. napus* × G210. By comparison, fewer KO modules were significantly enriched in the NTC treatment relative to *B. napus* × M26, indicating a greater degree of functional similarity between these two treatments.

Many of the functional modules enriched in NTC treatments included pathways for nitrogen cycling and sulfur assimilation (thiosulfate oxidation by SOX complex, sulfate-sulfur assimilation, and assimilatory sulfate reduction). Reduced representation of pathways involved in the cycling of inorganic compounds in treated samples might be explained by the high availability of organic forms in the seed meal. A number of modules enriched in DA KOs in NTC samples were also enriched in *B. napus* × M26 relative to BjSa (Additional file 2: Table S5). For example, Cobalamin (vitamin B12) biosynthesis was associated with both NTC and *B. napus* × M26 treatments, possibly pointing to a deficiency of this essential vitamin in the rhizosphere under these conditions.

KO accessions that were found to be more abundant in BjSa SM included such involved in the degradation and biosynthesis of the aromatic compounds toluene and dihydrokalafungin, respectively. It has been shown that bacteria containing toluene-degradative pathways can utilize a broad range of structurally similar substrates, including phenols [64]. Sinalbin (4-hyroxybenzyl glucosinolate), the main glucosinolate present in *S.alba* (a component of BjSa but not of *B. napus* SM), contains a phenol side-chain. Thus, it is likely that bacteria with the ability to degrade Toluene could potentially utilize sinalbin and Dihydrokalafungin as carbon substrates. These results point to unique biodegradative/bioremediative abilities specifically linked to *S. alba* SM. Beyond this specific example, functional capacities in many cases reflect unique environmental adaptations, in which the organic seed meal amendments can induce differences between the SM-treated and untreated control samples.

To interpret potential significance of specific DA functions beyond the identification of enriched pathways/modules, we focused on the characterization of the environmental resources that are associated with these reactions. Based on the metabolic networks formed by the sets of DA enzymes (enzymes that are DA in BjSa SM treated *vs* control samples, Fig. 1B), we applied computational approaches that predict externally sourced compounds representing a proxy of the relevant metabolic environment. Such compounds could potentially be derived from several sources, including SM, microbial secretion and plant exudates. We followed the process outlined in [29] for constructing metabolic networks and predicting environmental inputs that were unique to SM treated *vs* control samples (Fig. 1D). Reassuringly, pathways enriched in predicted source metabolites unique to the SM treated-samples (BjSa and *B. napus* × G210) included glucosinolates (Table 2, Additional file 2: Table S6), key components of the *Brassicaceous* SM mixtures [65]. Glucosinolates are sulfur and nitrogen-containing glycosides derived from amino acids that are found at high concentrations in Brassicaceous plants, particularly in the seeds [66, 67]. The presence of glucosinolates can be related to the prediction of excess sulfur and nitrogen in the SM-treated samples. Other unique environmental inputs enriched in SM-treatments were related to terpenoid metabolism, including many phytochemicals that can be found at high levels in Brassicaceous plants [67]. Overall, the network-based predictions of environmental inputs pointed at multiple compounds and phytochemicals associated with the source treatments (seeds of Brassicaceous plants) in the SM-treated samples but not in the control treatment. Predicted environmental resources enriched in NTC and/or *B.napus* × M26 treatments included a broad spectrum of compounds related to plant defense (e.g., phenylpropanoids, indole diterpene alkaloids and alkaloids derived from shikimate pathway). These types of compounds are likely to be elevated in root exudates of plants experiencing pathogenic pressure that can be expected in soil systems conducive to replant disease.

**Fig. 3:**
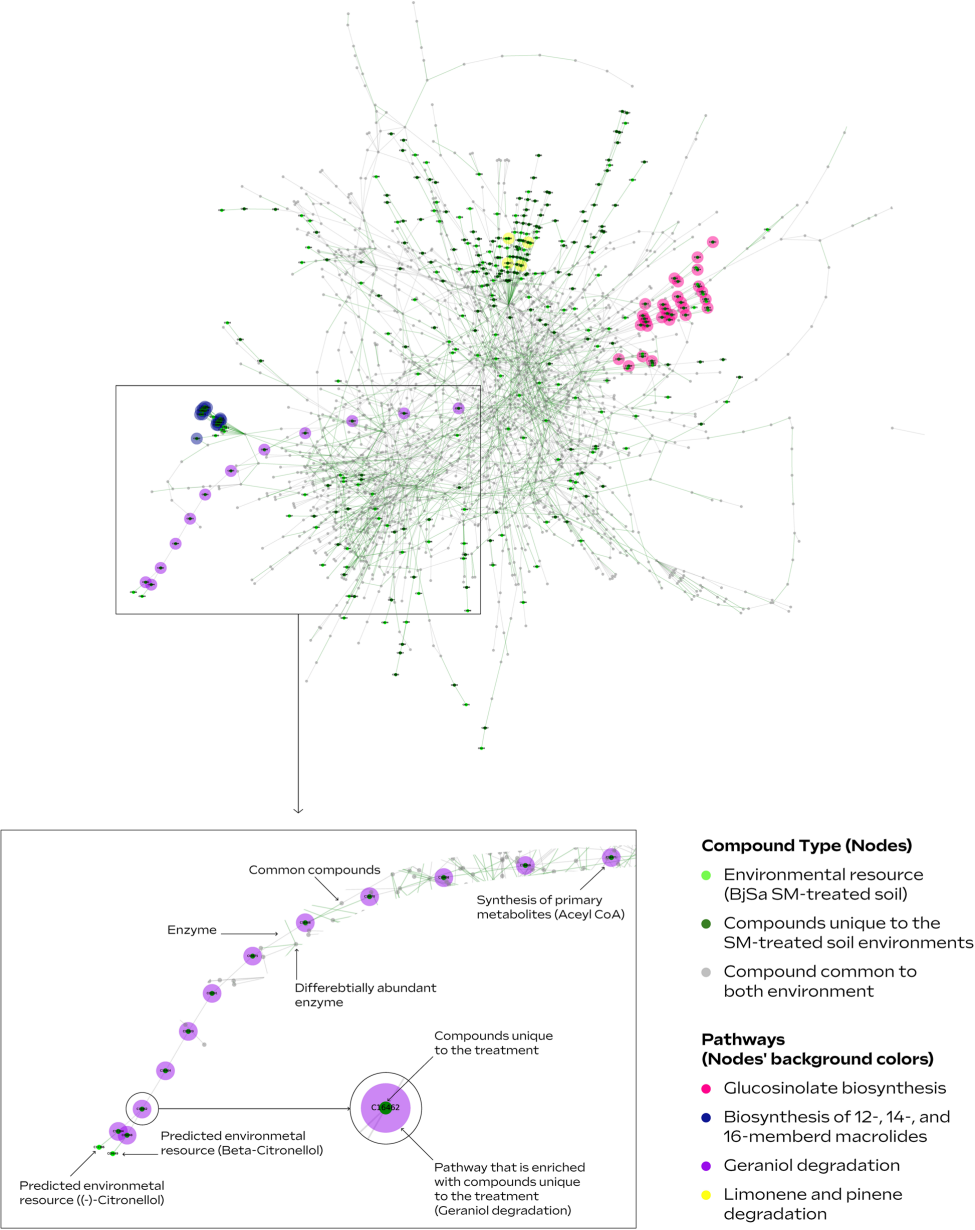
Visualization of the networks representing the metabolic activity in *Brassica juncea/Sinapis* alba seed meal (BjSa SM)-treated versus NTC (control) samples. Network was produced by simulating metabolic activities given a set of metabolic reactions (3060 enzymatic reactions) in BjSa SM-treated samples. Pathways that are enriched (FDR adjusted *P* value <  = 0.05) with network components (nodes) that are unique to the treated samples are indicated with nodes’ background color. A reciprocal image, visualizing the metabolic activity in NTC (control) versus BjSa SM-treated samples is provided in Additional file 2: Figure S7

Unlike the set of enzyme coding genes—reflecting the full functional potential of species in soil, actual metabolic performances are environment-dependent and reflect available nutritional sources. The predicted source-metabolites (environmental resources), together with the metabolic potential (the enzymes), allowed us to simulate metabolic activity in SM-treated *vs* control environments [27, 49] and explore the influence of environmental inputs on metabolic capacities *in a given environment*. Simulations generate a set of all possible metabolites that *can be* produced (representing “function”) given (1) a set of feasible reactions identified in the metagenome and (2) sets of compounds representing SM/NTC environments (Fig. 1F). The resulting networks represent the activity of the community in different samples (SM-treated *vs* control) and are composed of shared (grey) vs unique (colored) compounds (Fig. 3—SM-treated; Additional file 2: Figure S7). The high number of common compounds in BjSa SM and NTC samples (2004 compounds out of 2493 and 2340, respectively) can be expected in natural, robust systems where the majority of primary functions are conserved across multiple taxonomic groups and central metabolism is carried out despite environmental variations [68]. Visualization illustrates that whereas DA enzymes (colored network edges) are distributed across the metabolic networks, unique compounds (colored network nodes) can in many cases be grouped providing a clearer functional signature. Many of the pathways enriched in compounds unique to the BjSa SM involve the utilization of potential derivates of *Brassica* SM including glucosinolates and other potential components of oil-sourced seeds such as limonene [67], a volatile with antifungal activity [69], and geraniol with antimicrobial activity [70]. Finally, the enrichment patterns for these simulation-based metabolic networks (Additional file 2: Table S7) are in agreement with the enriched pathway profiles of DA enzymes and environmental resources (Table 2) in which functional pathways and metabolites unique to BjSa and *B. napus* × G210 vs. NTC largely differed from the enrichment profile of *B. napus* × M26 vs. NTC.

### Metagenomic stratification of microbial function: teasing out distinct taxa-function relationships

**Fig. 4:**
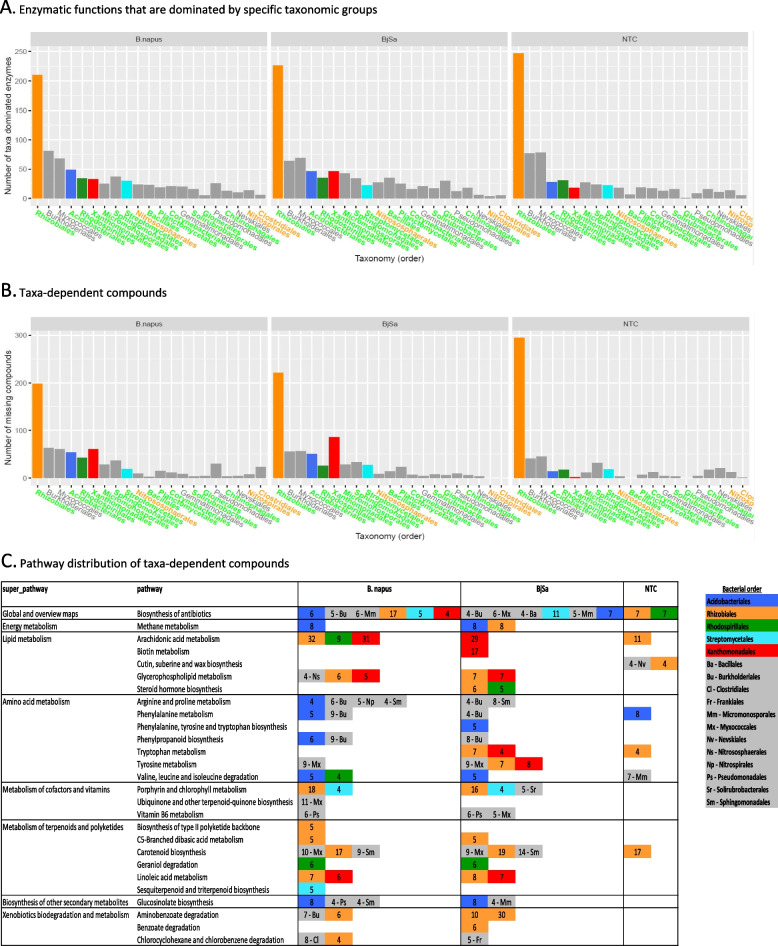
Distribution of order-level taxa-associated functions detected in the rhizosphere of G210 apple rootstock. Colored labels of taxonomic groups indicate significant abundance in the *Brassica juncea/Sinapis alba* seed meal (BjSa)SM treated (green) or control (orange) samples. Pathways with at least four taxa-dependent compounds are detailed. The distribution of genus-level taxa-associated functions is provided in Additional file 2: Figure S10

In addition to highlighting rhizosphere functions associated with recovery from replant disease, we further aimed to associate these functions with differentially abundant microbial groups (Fig. 1G). Enzymes were scored according to the taxonomic diversity of their associated reads (see “Methods” section). Taxa-dominance was determined for each enzyme according to the distribution of reads in specific samples and hence is treatment-specific rather than a constant attribute of the enzymes. For example, out of 3060 enzymes, 942, 904, and 814 were paired with a dominant taxa at the order-level and 661, 641, and 577 at the genus-level for BjSa SM × G210, *B. napus* SM × G210, and NTC × G210 treatments, respectively (Additional file 3). In accordance with the conservative nature of metabolism [71], the majority of enzymatic functions were found to be carried by most community members. Nonetheless, our analysis suggested that specific enzymatic functions are dominantly carried (according to approximation) by distinct taxonomic groups (Fig. 4A). In some cases, taxonomic groups that were differentially abundant in SM treatment (green) *vs* NTC (orange) had a higher number of signature enzymes in the respective sample (e.g., *Bacillus* and *Glomerales* in BjSa and Candidatus Nitrosolenus in NTC samples; Fig. 4A). The taxa-dominated enzymes mostly mapped to multiple metabolic pathways with no clear taxonomic signature across specific functions (Additional file 2: Table S8).

**Fig. 5:**
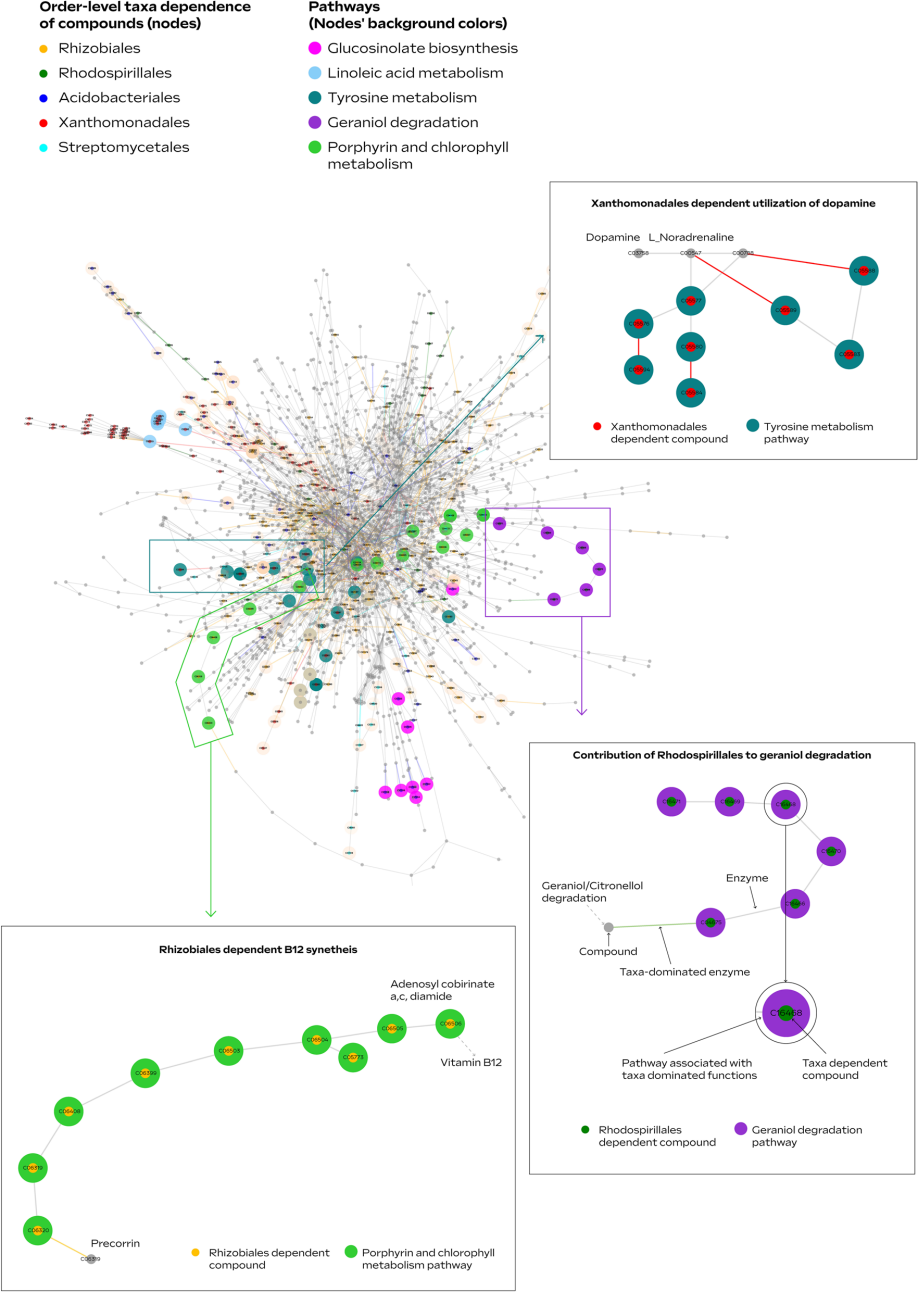
Taxonomic stratification of microbial functions. Visualization of order-level dominated functions (enzymes and compounds) in a network representing the overall metabolic activity in Brassica juncea/Sinapis alba seed meal (BjSa) treated soil. Colored edges and nodes in the network represent taxa-dominated enzymes and taxa-dependent compounds in BjSa X G210, respectively, shown here at the order level and in Additional file 2: Figure S8 and Additional file 2: Table S10 at the genus level. The nodes’ background color indicate the compounds associated with the pathways presented in Fig. 4

In order to boost the signal for potential functional roles of key (e.g., differentially abundant) taxonomic groups *in the treated samples* (highlighted in Fig. 4), we carried out iterative simulations of ‘knock out’ microbiomes considering the environmental resources characteristic of the treatment. Simulations were carried out using the predicted environmental resources and set of reactions used to describe activity in the treatment/control samples, while conducting iterative eliminations of groups of taxa-dominated enzymes, one group at a time (illustrated in Fig. 1G). The impact of the removal of each key taxonomic group was estimated according to differences in the metabolite content between the original meta-network, expanded from the full enzymatic set (Fig. 3), and the network expanded from the truncated enzyme set (Fig. 5). The key difference between network-based activity simulations (as shown in Figs. 3 and 5) vs static functional characterization such as pathway completeness (as carried out in many genome centric metagenomics analyses [72]) is that the simulations reflect the robustness of the network to function/enzyme removal and the effect of the environment. That is, pathway completeness analysis cannot reflect the hierarchical positioning of a reaction in a pathway and its redundancy (the prospects of finding alternative routes for producing the corresponding metabolites). For example, an enzyme converting a predicted source-metabolite into a compound accessible by multiple groups in the community will have a high impact in network-based simulations, particularly if there are no alternative biosynthetic pathways, but not in pathway-completeness analysis. In addition, simulations are environment-specific; hence, as in natural ecosystems, the functional impact of each taxonomic group is environment-dependent. Despite the robustness of the expanded metabolic networks, removing all enzymes dominated by taxonomic groups at the genus and order level led to 21% and 31% reduction in network size, respectively, when omitting all of the taxa-dominated enzymes together.

The number of taxa-dominated enzymes (Fig. 4A) was in general agreement with the number of taxa-dependent compounds, defined as compounds that were eliminated from the network following the knock-out of corresponding groups in the simulation (Fig. 4B, Additional file 9). Pathway distribution of taxa-dependent compounds suggested unique functional signatures of key groups (Fig. 4C, Additional file 2: Table S8), possibly associated with a direct or indirect effect on the ‘recovery’ phenotype of the orchard. For example, antibiotics such as Albaflavenone (KEGG accession C17954) and its precursor Epi-isoizaene (C16269) were predicted as Streptomycetales taxa-dependent compounds in accordance with biochemical reports [73]. Streptomycetales were highly abundant in SM-treated samples and their unique profile of antibiotics might lead to the specific suppression of some of the under-represented groups. Myxococcales (highly abundant in the *B. napus* SM treated samples), another order associated with unique contribution to the biosynthesis of antibiotics, are also known to be suppressors of root microbial pathogens with antibacterial and antifungal activities [74, 75]. Other literature supported examples included the contribution of Sphingomonadales to *carotenoid* metabolism [76] and of Xanthomonadales to linoleic acid metabolism [77]. Linoleic acids are natural inhibitors of nitrification in soil, and were shown to act as suppressors of *Nitrosomonas* [78], a genus that was under-represented in the BjSa SM amended samples in comparison to the control (Additional file 3). *Mycobacterium*, predicted to have a unique contribution in steroid hormone metabolism, are known to have the capacity of utilizing sterols as a carbon source [79]. Some of the functional categories associated with the SM treatment (Fig. 3) can be related to specific groups that are differentially abundant in these samples (Fig. 5): *Bradyrhizobium* were associated with Limonene and pinene metabolism; Rhodospirillales (mainly its genus *Reyranella*) were associated with geraniol degradation; Acidobacteriales (mainly Candidatus Koribacter) have unique contribution to glucosinolate metabolism, possibly reflecting the ability of this taxon to exploit organic sulfur molecules for energy conversion [80, 81].

### Prediction and testing of the effect of specific metabolites as biostimulants/biosuppressors of beneficial taxonomic groups

The ultimate goal of the simulations described above was to highlight specific compounds that may be beneficial to organisms contributing to disease control, or deleterious to organisms contributing to disease progression. To this end, we screened for (i) compounds whose *utilization* depends on specific microbial groups, hence can act as taxa-specific biostimulants or (ii) compounds that serve as essential metabolites for many taxa through exchange interactions, but whose *production* depends on specific microbial groups [82, 83]. Dominance in producing an essential metabolite may convey a fitness advantage to the producer if the compound is in limited supply; an advantage may be lost when the compound is in excess. Hence, such compounds may act as taxa-specific biosupressors. Adding such compounds to soil could lead to a predicted effect on community structure associated with the disease recovery phenotype.

For the design of experimental validation, the limited collection of treatment-specific, taxa-dependent compounds (Fig. 4), was scrutinized via a review of the literature (Additional file 2: Table S9). A short list of potential biostimulants and biosupressors was further limited by considering only those metabolites that can be added to soil in view of regulatory and commercial aspects. The selected metabolites included dopamine and vitamin B12 as potential biostimulants (scenario i) and biosuppressors (scenario ii) of beneficial taxa, respectively. Dopamine is a non-toxic catecholamine, a group of compounds detected in many *Brassica* plants [84] and was shown to enhance the growth of a variety of Proteobacteria [51, 85]. In the BjSa SM-treated samples (and not NTC), metabolism of catecholamine derivatives (included in the KEGG pathway of tyrosine metabolism) was predicted to depend on bacterial taxa within the order Xanthomonadales (Fig. 5). This proteobacterial group was highly-abundant in the treated samples and is likely to contain beneficial members associated with suppression of replant fungal and oomycete pathogens [86, 87]. We hypothesized that the addition of dopamine to BjSa SM-amended soil would increase the relative abundance of Xanthomonadales groups which can metabolize catecholamine. In both SM-treated samples, synthesis of the co-factor vitamin B12 (included in the KEGG category of Porphyrin and Chlorophyll metabolism) was predicted to be dependent on bacteria within the orders Rhizobiales and Streptomycetales (Fig. 5). Both of these groups were recently identified as key producers of vitamin B12 in soil [53, 88–90]. We hypothesized that the addition of vitamin B12 to the BjSa SM soil system would reduce the producers’ competitive advantage in the rhizosphere compared to soil treated with BjSa SM alone.

**Fig. 6:**
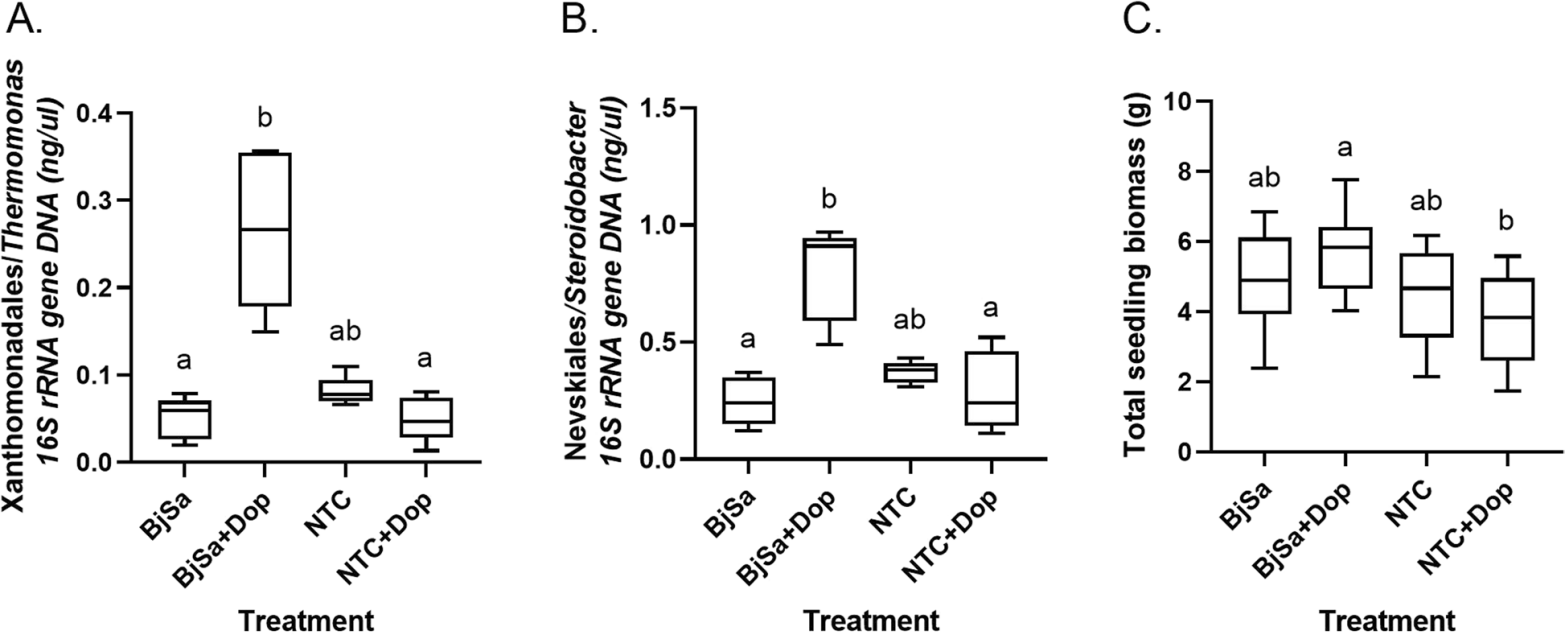
Testing of the effect of dopamine as a biostimulant of beneficial taxonomic groups**.** Box-and-whiskers plots show the abundance of select bacterial genera within the orders Xanthomonadales (**A**) and Nevskiales (**B**), predicted by network analysis to be linked to the utilization of dopamine and seedling biomass measured upon harvest (8 weeks post-planting; *n* = 10) (**C**). 16S rRNA gene sequence data was obtained from DNA extracted from rhizosphere soil collected 4 weeks post-planting into the respective treatments (*n* = 5). Values plotted represent the 25th percentile, the median, the 75th percentile; whiskers extend to the minimum and maximum. In **A** and **B**, multiple comparisons were carried out with Dunn’s test following the Kruskal–Wallis non-parametric test. In **C**, multiple comparisons were performed using Tukey’s test following ordinary one-way ANOVA. Unlike the original experiment, seedling biomass was similar between BjSa SM and NTC

The predicted effects of the potential biostimulant (dopamine) and biosuppressor (vitamin B12) metabolites were tested in a four-condition experimental pot system, adhering to the methods utilized in the original experiment: BjSa SM treatment, BjSa SM treatment with supplement (dopamine or vitamin B12), control (NTC), control with supplement. As expected from the network-based predictions, the relative abundance of several OTUs belonging to the Xanthomonadales were significantly higher in the BjSa + dopamine treatment relative to both BjSa SM alone and NTC + dopamine (Fig. 6A; *p* = 0.0018 and *p* = 0.0164, respectively), including two taxa (*Dokdonella* spp. and *Thermomonas dokdonensis*) which were positively correlated with root biomass (Additional file 2: Table S10).

In terms of tree performance, seedling biomass was greatest in BjSa SM + dopamine treated soils, though a significant difference in biomass was detected only between BjSa SM + dopamine and NTC + Dopamine treatments (highest and lowest, respectively, Fig. 6C; *p* = 0.025). The opposing effects of dopamine on BjSa SM-amended vs. NTC treatments can be explained by the impact of the SM on the taxonomic composition and metabolic activity of the soil microbiome before dopamine addition. In addition to enrichment of the Xanthomonadales groups, a significant increase in the relative abundance of species from the Nevskiales order, primarily belonging to the Sinobacteraceae family, was observed in BjSa SM + dopamine relative to BjSa (Fig. 6B; *p* = 0.0048). The Nevskiales, particularly the Sinobacteraceae family, is closely related and taxonomically-tangled with Xanthomonadales [91]. In the G210 samples, the network-based analysis pointed at dominance of the order Xanthomonadales in metabolizing catecholamine, whereas the Nevskiales were identified as the dominant group carrying out the same function in M26 samples (Additional file 2: Figure S8).

Unlike our hypothesis, adding Vitamin B12 to BjSa SM-amended soil did not result in a reduction of groups from either Rhizobiales or Streptomycetales orders. In accordance with no significant change in the relative abundance of these beneficial taxa, plant performances were not affected by the B12 treatment (Additional file 2: Figure S10). One possible reason for the lack of differences in the response of predicted producer taxa to vitamin B12 addition may be related to the complexity and high costs of its production. Thus, when cobalamin is abundant, producers may switch from synthesis to salvage pathways providing them a competitive advantage.

## Conclusions

The primary goal of the study reported here was to garner an understanding of metabolites instrumental in directing the assembly of a disease-suppressive microbiome (either directly from the SM amendment or through the induction of specific taxa that secrete those compounds). Considering the network complexity of the rhizosphere microbiome, the art of strategically engineering microbial communities to optimize functions of interest is extremely challenging and has not yet achieved a reliable predictability. However, the use of integrated approaches relying on genomics/metagenomics-based documentation of community structure and dynamics, together with metabolic modeling approaches, is rapidly paving the way for the systematic description of ecosystem architecture. The development of this knowledge base can then be used to promote the design of effective resource management schemes to harness the potential of the indigenous microbiome towards pre-defined functions [22, 25, 31, 32, 92]. This work demonstrates the formulation of model-based predictions from network analyses of metagenomics data and the subsequent testing performed to validate those predictions. The gene-centric approach taken here is inclusive in the sense that unlike many recent studies that focus on fully recovered genomes, it allows for the representation of less abundant species, with almost 70% of the reads being included—a critical aspect for the study of a the highly diverse ecosystem such as soil. This gene-centric approach for data analysis resulted in a three-fold increase in the number of identified genera in comparison to those identified by amplicon sequencing. The projection of taxa-dominated functions over the meta-network, representing a composite collection of the complement set of enzymes in soil, provides an overview of core vs specialized functions. This community view of metabolism reflects the recognition that metabolic exchanges serve as pivotal determinants of the structure and function of ecosystems in general and of microbial communities in particular [26, 82, 93, 94]. Using this gene-centric approach, we were able to formulate hypotheses for compounds that serve as modulators of microbial composition in the rhizosphere. As a case study, experimental validation of the compound dopamine corroborated its predicted role as modulator of specific plant-beneficial taxa. However, the development of conclusive protocols for the active manipulation of plant microbiomes will require more detailed exploration of the predictions as well as careful calibration of application rates and timing. Moreover, the differential effects of dopamine on the abundance of Xanthomonadales in BjSa SM and NTC treatments highlights the impact of the intricate network of interactions in soil where multiple factors determine species’ abundance. Hence, future development of a more robust validation system will likely rely on a combination of culture-dependent and independent methods. For example, recovery of metagenome-assembled genomes (MAGs) or even strain-resolved genomes [58] could be used to predict growth conditions which would allow for selective cultivation of biologically functional strains. In addition, sequence data could be used to develop qPCR methodologies that specifically target the suspected organism(s) and/or functional gene(s) in order to quantify the relative occurrence in the roots and/or soil.

The generation of testable predictions based on the interpretation of genomic data is a first step towards untangling the intricate and dynamic web of microbial interactions in soil. To date, few soil amendment-based protocols have demonstrated the consistent and effective performance required to supplant conventional disease control practices, such as soil fumigation. The application of data-driven predictions targets trial-and-error protocols which enable development of optimal solutions for disease control (in this case, the use of organic amendments to recruit indigenous microorganisms possessing diverse functions including the capacity to suppress high pathogen loads). The framework used here for generating predictions for the selective targeting of microbial groups based on processing assembled and annotated metagenomics data is available as a pipeline at https://github.com/ot483/NetCom2.

Future metagenomic analyses may integrate additional data layers, including metabolomics, transcriptomics, and the recovery of complete genomes. The applications of these analytical tools in experimental design go well beyond the specific model suggested in this study and can be easily applied to many other cropping systems. In fact, such a platform is broadly applicable to harnessing plant—microorganism and microorganism—microorganism interactions in many different environmental scenarios (e.g., remediation of contaminated sites).

The optimization of predictable outcomes from such a strategy has the potential to enable a reduction in agrochemical use. Restoring a balance between the needs of a constantly growing population and protecting the environment for future generations poses a significant challenge to developing ecologically sustainable solutions. In an era of ecosystem degradation and climate change, optimization of microbiome function in agroecosystems offers one of the few untapped routes to reducing reliance on agrochemicals and restoring the health of the soil microbiome [95, 96]_._

## Supplementary Information

**Additional file 1.** A summary of the number of sequenced reads per library.

**Additional file 2.** Supplementary Tables and figures.

**Additional file 3.** Differentially abundant taxonomic groups (genus and order levels).

**Additional file 4.** Differentially abundant functional groups (KO & EC functional schemes).

**Additional file 5.** Lists of taxa-dominated enzymes. Analysis is based on samples from treatments with G210 apple rootstocks.

**Additional file 6.** Topological properties of enzymes (network degree) vs their score of taxonomic dominance. Analysis is based on samples from treatments with G210 apple rootstocks.

**Additional file 7.** Predicted environmental resources and network specific compounds in BjSa vs NTC.

**Additional file 8.** Lists of taxa-dependent compounds. Analysis is based on samples from treatments with G210 apple rootstocks.

**Additional file 9.** Distribution of the number of reads, taxa-dominated enzymes and taxa-dependent compounds across taxonomic groups in different treatments. The analysis is based on samples from treatments with G210 apple rootstocks.

## Data Availability

A detailed reproducible bioinformatics workflow for all the computational analyses was deposited in https://github.com/ot483/NetCom2. Raw metagenome sequences have been deposited in NCBI under BioProject accession number PRJNA779554 (https://www.ncbi.nlm.nih.gov/bioproject/PRJNA779554). Other data files analyzed during this study are included in this published article and its supplementary files.
