# Supplementary material for: A framework for the targeted recruitment of crop-beneficial soil taxa based on network analysis of metagenomics data
Source: Microbiome. 2023 Jan 12;11:8. doi: 10.1186/s40168-022-01438-1 (PMC9835355; doi:10.1186/s40168-022-01438-1)
Supplement: Supplementary file 3 — Additional file 2. Supplementary Tables and figures. [file 40168_2022_1438_MOESM2_ESM.docx]

**Supplementary Tables and Figures**

**A framework for the targeted recruitment of crop-beneficial soil taxa based on network analysis of metagenomics data**

**List of Content**

**Supplementary Tables 1-10**

**Supplementary Figures 1-10**

**Supplementary Table 1.** Assembly statistics. Each library is consistent of five replicates. ¥contigs > 2000 bp

| Library | #contigs | #contigs>  2000 bp | #bp¥ | average  bp/contig¥ | N50¥ | Percentage of reads  mapped¥ | #protein  coding genes¥ |
| --- | --- | --- | --- | --- | --- | --- | --- |
| BjSa_G210 | 22761272 | 1490790 | 7090901141 | 4756 | 5272 | 71 | 7701991 |
| BjSa_M26 | 28816496 | 1955528 | 9185158914 | 4697 | 5155 | 73 | 10078846 |
| *B.napus* _G210 | 26189227 | 1424830 | 6351700797 | 4458 | 4742 | 63 | 6927470 |
| *B.napus* _M26 | 25773948 | 1497399 | 6775561351 | 4525 | 4850 | 65 | 7335882 |
| NTC_G210 | 27622041 | 1464194 | 6531352559 | 4461 | 4756 | 61 | 7079648 |
| NTC_M26 | 33996648 | 1884536 | 8517471499 | 4520 | 4860 | 63 | 9215716 |

**Supplementary Table 2.** Annotation statistics.

| Library | #protein  coding genes | Percentage of proteins with taxonomic annotations | | | | | Percentage of proteins with functional annotations | | |
| --- | --- | --- | --- | --- | --- | --- | --- | --- | --- |
|  |  |  |  |  |  |  |  |  |  |
|  |  | Domain level | Phylum level | Order level | Genus level | Species level | Seed | KEGG_(KO) | KEGG_EC |
| BjSa_G210 | 7701991 | 57 | 46 | 31 | 21 | 15 | 74 | 70 | 16 |
| BjSa_M26 | 10078846 | 57 | 46 | 31 | 21 | 15 | 56 | 54 | 13 |
| *B.napus*_G210 | 6927470 | 57 | 44 | 30 | 21 | 15 | 82 | 79 | 19 |
| *B.napus* _M26 | 7335882 | 58 | 44 | 30 | 21 | 15 | 78 | 50 | 12 |
| NTC_G210 | 7079648 | 57 | 42 | 28 | 20 | 15 | 82 | 50 | 12 |
| NTC_M26 | 9215716 | 57 | 43 | 29 | 20 | 15 | 62 | 39 | 9 |

**Supplementary Table 3**. Percentage of contig-level taxonomic assignments carried out by MEGAN**.**

| **Treatment** | **Megan annotations contigs** | |  |
| --- | --- | --- | --- |
|  | Order (number of contigs) | Genus (number of contigs) | |
| BjSa_G210 | 96 (1415056) | 94 (1382568) | |
| BjSa_M26 | 96 (1856601) | 94 (1813213) | |
| *B.napus* _G210 | 96 (1356650) | 94 (1330280) | |
| *B.napus* _M26 | 97 (1442778) | 96 (1429250) | |
| NTC_G210 | 96 (1393197) | 94 (1366340) | |
| NTC_M26 | 96 (1793694) | 94 (1758343) | |

**Supplementary Table 4.** Taxonomic and functional groups identified based on shotgun metagenomics and amplicon data. Amplicon data was taken from Somera et al 2021[^1^](#_heading=h.30j0zll).

| **Treatment** | **Shotgun metagenomics** | | | | **Amplicon** | |
| --- | --- | --- | --- | --- | --- | --- |
|  | Phylum | Order | Genus | EC | Phylum | Genus |
| BjSa_G210 | 119 | 275 | 1740 | 2814 | 25 | 682 |
| BjSa_M26 | 117 | 287 | 1856 | 2901 | 26 | 716 |
| *B.napus* _G210 | 114 | 274 | 1720 | 2763 | 27 | 754 |
| *B.napus* _M26 | 169 | 240 | 1778 | 2668 | 27 | 761 |
| NTC_G210 | 118 | 282 | 1741 | 2587 | 26 | 773 |
| NTC_M26 | 121 | 309 | 1849 | 2722 | 27 | 790 |

**Supplementary Table 5.** KEGG modules significantly enriched with DA KO accessions. Processes that are enriched in NTC (control) entities are colored in orange; processes that are enriched in BjSa SM treatment entities are colored in green; processes that are enriched in *B. napus* SM treatment entities are colored in blue. Dark coloring indicates significance (FDR adjusted P value <= 0.05); bright coloring indicates the experimental conditions with which the majority of entities are associated (thought below significance threshold). Only pathways with at least six entities with clear dominance (>=75%) of one of the treatments and exhibiting significance in at least one of the comparisons tested are shown. NA denotes pathways with less than six entities assigned.

| **B.napus vs BjSa** | | **B.napus vs NTC** | | **BjSa vs NTC** |  |
| --- | --- | --- | --- | --- | --- |
| **M26** | **G210** | **M26** | **G210** |  |  |
| 1894/3040 | 1702/2036 | 1029/1399 | 2215/1973 | 3056/2193 | Total KO (7741) |
| 392/537 | 358/320 | 175/277 | 483/421 | 547/484 | Assigned to modules |
| 6/6 | 6/6 | 4/4 | 6/6 | 6/6 | Thiosulfate oxidation by SOX complex, thiosulfate => sulfate |
| 11/13 | NA | 6/7 | 11/12 | 11/14 | Sulfate-sulfur assimilation |
| NA | NA | NA | 7/7 | 7/8 | Dissimilatory nitrate reduction, nitrate => ammonia |
| NA | NA | NA | 7/8 | 6/7 | Denitrification, nitrate => nitrogen |
| 7/8 | NA | NA | 8/8 | 8/9 | V/A-type ATPase, prokaryotes |
| 7/9 | NA | NA | 7/8 | 7/10 | Assimilatory sulfate reduction, sulfate => H2S |
| 5/6 | 6/7 | 5/5 | 4/6 | 6/7 | Glycogen biosynthesis, glucose-1P => glycogen/starch |
| NA | NA | 4/4 | NA | 5/6 | ADP-L-glycero-D-manno-heptose biosynthesis |
| NA | NA | NA | 6/7 | 6/7 | C5 isoprenoid biosynthesis, mevalonate pathway, archaea |
| NA | NA | 2/4 | NA | 6/7 | Nicotinate degradation, nicotinate => fumarate |
| 6/7 | NA | NA | 6/7 | 5/7 | Cobalamin biosynthesis, cobinamide => cobalamin |
| NA | NA | NA | 5/6 | 7/7 | C5 isoprenoid biosynthesis, non-mevalonate pathway |
| NA | NA | 5/6 | NA | 8/8 | Undecylprodigiosin biosynthesis, L-proline => undecylprodigiosin |
| 6/6 | NA | NA | NA | 6/6 | Toluene degradation, anaerobic, toluene => benzoyl-CoA |
| 6/6 | 4/6 | NA | NA | 6/6 | Dihydrokalafungin biosynthesis, octaketide => dihydrokalafungin |
| 6/6 | NA | NA | NA | 7/7 | Malonate semialdehyde pathway, propanoyl-CoA => acetyl-CoA |
| NA | NA | 4/5 | NA | 7/7 | Prodigiosin biosynthesis, L-proline => prodigiosin |
| NA | NA | NA | 6/6 | 7/7 | Propanoyl-CoA metabolism, propanoyl-CoA => succinyl-CoA |
| 9/9 | NA | 6/6 | 7/10 | 3/6 | Xanthomonas spp. pathogenicity signature, T3SS and effectors |

| **B.napus Vs BjSa** | | **B.napus Vs NTC** | | **BjSa Vs NTC** |  |
| --- | --- | --- | --- | --- | --- |
| **G210** | **M26** | **G210** | **M26** |  |  |
| 224/170 | 218/347 | 318/293 | 118/137 | 152/205 | Total seeds (790) |
| 163/110 | 145/219 | 210/197 | 95/93 | 229/190 | Assigned to pathway |
| NA | NA | 9/9 | NA | 9/10 | Steroid biosynthesis |
| NA | NA | 6/6 | NA | 6/6 | Indole diterpene alkaloid biosynthesis |
| 4/6 | 5/6 | 7/8 | NA | 7/7 | Naphthalene degradation |
| 11/12 | 8/13 | NA | 11/11 | NA | Biosynthesis of alkaloids derived from shikimate pathway |
| 24/32 | 30/50 | 23/40 | 20/25 | 25/45 | Biosynthesis of antibiotics |
| NA | 6/7 | 9/11 | 7/7 | 9/17 | Chlorocyclohexane and chlorobenzene degradation |
| NA | 3/6 | 8/8 | 4/6 | 7/9 | Phenylpropanoid biosynthesis |
| NA | 10/14 | 11/11 | NA | 11/15 | Biosynthesis of unsaturated fatty acids |
| NA | 11/17 | 13/13 | 3/6 | 12/16 | Fatty acid metabolism |
| 7/10 | 3/6 | 9/11 | NA | NA | Ubiquinone and other terpenoid-quinone biosynthesis |
| 10/10 | 7/9 | 6/6 | NA | 6/8 | Biosynthesis of 12-, 14- and 16-membered macrolides |
| 7/7 | NA | 11/11 | NA | N/A | Steroid hormone biosynthesis |
| 6/6 | 12/12 | 12/12 | NA | 12/12 | Glucosinolate biosynthesis |
| NA | 6/6 | NA | NA | 6/6 | Purine metabolism |
| 3/6 | 8/8 | 7/7 | NA | 6/6 | Biosynthesis of terpenoids and steroids |
| 5/9 | 6/6 | NA | NA | 7/7 | Cyanoamino acid metabolism |
| NA | NA | NA | NA | 7/7 | Chloroalkane and chloroalkene degradation |
| NA | 7/7 | NA | NA | 7/7 | Monoterpenoid biosynthesis |
| 6/6 | 12/12 | 12/12 | NA | 12/12 | 2-Oxocarboxylic acid metabolism |
| 7/7 | 6/11 | 6/11 | NA | 6/11 | Anthocyanin biosynthesis |
| 12/14 | 9/17 | 8/15 | 7/9 | 9/17 | Biosynthesis of type II polyketide products |

**Supplementary Table 6.** KEGG pathways significantly enriched with treatment-specific environmental resources. Enriched pathways are such that have at least six entities assigned and a clear dominance of one of the treatments (>=80%) of one of the treatments. Processes that are enriched in NTC (control) entities are colored in orange; processes that are enriched in BjSa SM treatment entities are colored in green; processes that are enriched in *B. napus* SM treatment entities are colored in blue. Dark coloring indicates significance (FDR adjusted P value <= 0.05); bright coloring indicates the experimental conditions with which the majority of entities are associated (thought below significance threshold). Only pathways with at least six entities with clear dominance (>=75%) of one of the treatments and exhibiting significance in at least one of the comparisons tested are shown. NA denotes pathways with less than six entities assigned.

**Supplementary Table 7.** KEGG pathways significantly enriched with treatment-specific compounds (network expanded metabolites). Enriched pathways are such that have at least six entities assigned and a clear dominance of one of the treatments (>=80%) of one of the treatments. Processes that are enriched in NTC (control) entities are colored in orange; processes that are enriched in BjSa SM treatment entities are colored in green; processes that are enriched in *B. napus* SM treatment entities are colored in blue. Dark coloring indicates significance (FDR adjusted P value <= 0.05); bright coloring indicates the experimental conditions with which the majority of entities are associated (thought below significance threshold). Only pathways with at least six entities with clear dominance (>=75%) of one of the treatments and exhibiting significance in at least one of the comparisons tested are shown. NA denotes pathways with less than six entities assigned.

| **B.napus  vs NTC** | | **BjSa vs NTC** |  |
| --- | --- | --- | --- |
| M26 | G210 |  |  |
| 252/229 | 441/413 | 489/336 | Total Compounds |
| 216/184 | 332/355 | 387/294 | Assigned to pathway |
| NA | 33/33 | 33/33 | Glucosinolate biosynthesis |
| NA | 21/21 | 21/23 | Biosynthesis of 12-, 14- and 16-membered macrolides |
| NA | 15/15 | 13/13 | Geraniol degradation |
| NA | 19/23 | 11/11 | Limonene and pinene degradation |
| 7/7 | NA | 10/10 | Chloroalkane and chloroalkene degradation |
| 12/12 | NA | 12/13 | Fluorobenzoate degradation |
| 25/25 | 12/23 | 28/37 | Chlorocyclohexane and chlorobenzene degradation |
| 10/11 | 10/11 | 10/11 | Caffeine metabolism |
| 12/12 | 18/18 | 18/18 | Metabolism of xenobiotics by cytochrome P450 |
| 16/16 | 14/14 | 15/16 | Steroid biosynthesis |
| 6/9 | 12/17 | 12/12 | Naphthalene degradation |
| NA | 20/22 | 14/15 | Ubiquinone and other terpenoid-quinone biosynthesis |
| 8/8 | 17/17 | 12/14 | Phenylpropanoid biosynthesis |
| 26/36 | 26/32 | 25/37 | Polycyclic aromatic hydrocarbon degradation |
| 10/10 | 19/19 | 13/17 | Xylene degradation |
| 4/6 | 14/14 | 12/16 | Biosynthesis of unsaturated fatty acids |
| 7/10 | 13/16 | 13/19 | Drug metabolism - cytochrome P450 |
| NA | 12/14 | 5/7 | Steroid hormone biosynthesis |
| 8/8 | 11/14 | 11/14 | Bisphenol degradation |
| 36/45 | NA | NA | Biosynthesis of antibiotics |
| 15/17 | 16/32 | 18/34 | Biosynthesis of type II polyketide products |
| 13/16 | 5/7 | NA | Tryptophan metabolism |
| 6/6 | NA | NA | Biosynthesis of ansamycins |
| 7/7 | NA | NA | Vitamin B6 metabolism |
| 14/18 | 12/16 | 7/11 | Toluene degradation |

**Supplementary Table 8. Pathway distribution of taxa-dominated enzymes.** Analyses are based on samples from G210 rootstocks.

**a.**

| **Genus** |  |  |  |
| --- | --- | --- | --- |
|  |  |  |  |
|  | **BjSa** | ***B. napus*** | **NTC** |
| **KEGG pathway** |  |  |  |
| Biosynthesis of antibiotics | 7 | 8 | 9 |
| Arginine and proline metabolism | 5 |  |  |
| Aminobenzoate degradation |  | 6 |  |
| Porphyrin and chlorophyll metabolism |  | 9 | 8 |
|  |  |  |  |
| Streptomyces |  |  |  |
| Sphingomonas |  |  |  |
| Bradyrhizobium |  |  |  |

**b.**

| **Order** |  |  |  |  |  |  |  |  |  |  |  |
| --- | --- | --- | --- | --- | --- | --- | --- | --- | --- | --- | --- |
|  |  |  |  |  |  |  |  |  |  |  |  |
|  | **BjSa** | | | | ***B. napus*** | | | | **NTC** | | |
| **KEGG pathway** |  | | | |  | | | |  | | |
| Amino sugar and nucleotide sugar metabolism | 5 |  |  |  |  |  |  |  | 5 |  |  |
| Aminobenzoate degradation | 7 |  |  |  | 8 |  |  |  | 13 |  |  |
| Benzoate degradation | 10 |  |  |  |  |  |  |  | 13 |  |  |
| Biosynthesis of antibiotics | 6 | 11 | 9 | 7 | 7 | 6 | 8 | 5 | 12 | 12 | 8 |
| C5-Branched dibasic acid metabolism | 6 |  |  |  | 6 |  |  |  | 6 |  |  |
| Carbon fixation pathways in prokaryotes |  |  |  |  | 5 |  |  |  | 6 |  |  |
| Cysteine and methionine metabolism | 5 |  |  |  |  |  |  |  |  |  |  |
| Glyoxylate and dicarboxylate metabolism | 10 |  |  |  | 9 |  |  |  | 7 |  |  |
| Methane metabolism | 8 |  |  |  | 6 |  |  |  | 7 |  |  |
| Phenylalanine metabolism |  |  |  |  |  |  |  |  | 7 |  |  |
| Porphyrin and chlorophyll metabolism | 7 |  |  |  | 11 |  |  |  | 12 |  |  |
| Sesquiterpenoid and triterpenoid biosynthesis | 5 |  |  |  |  |  |  |  |  |  |  |
| Steroid biosynthesis | 5 |  |  |  |  |  |  |  |  |  |  |
| Terpenoid backbone biosynthesis | 5 |  |  |  | 5 |  |  |  | 5 |  |  |
| Xylene degradation |  |  |  |  | 5 |  |  |  |  |  |  |
|  |  |  |  |  |  |  |  |  |  |  |  |
| Rhizobiales |  |  |  |  |  |  |  |  |  |  |  |
| Myxococcales |  |  |  |  |  |  |  |  |  |  |  |
| Micromonosporales |  |  |  |  |  |  |  |  |  |  |  |
| Streptomycetales |  |  |  |  |  |  |  |  |  |  |  |
| Acidobacteriales |  |  |  |  |  |  |  |  |  |  |  |

**Supplementary Table 9.** Selected examples of SM treatment-specific, functional predictions linked to specific taxa (order level). Experimentally tested predictions are shown in green.

| **Support/Possible interpretation** | **Function type** | **Function** | **Taxonomic group** |
| --- | --- | --- | --- |
| Catecholamines were shown to enhance the growth of Proteobacteria [^2^](#_heading=h.2s8eyo1). | Utilization of environmental resources | Metabolism of catecholamines (Tyrosine derivate) | Xanthomonadales |
| Rhizobiales are known to produce Vitamin B12 in soil[^3^](#_heading=h.17dp8vu). | Production of essential metabolites | Production of Vitamin B12 | Rhizobiales and Streptomycetales |
| Myxococcales are known to suppress root microbial pathogens with antibacterial and antifungal activities[^4^](#_heading=h.3rdcrjn). Streptomycetales are producers of multiple antibiotics. | Production of biocontrol metabolites | Biosynthesis of antibiotics | Streptomycetales, Myxococcales |
| Most *Sphingomonadales* species produce carotenoids[^5^](#_heading=h.26in1rg). | Production of other 2° metabolites | Carotenoid biosynthesis | *Sphingomonadales* |
| Xanthomonadales are known to contribute to linoleic acid metabolism[^6^](#_heading=h.lnxbz9). | Production of biocontrol metabolites | Linoleic acid metabolism | Xanthomonadales |
| May reflect the ability of this taxon to exploit organic sulfur molecules for energy conversion[^7^](#_heading=h.35nkun2)^,^ [^8^](#_heading=h.1ksv4uv). | Utilization of environmental resources | Glucosinolate metabolism | Acidobacteriales |


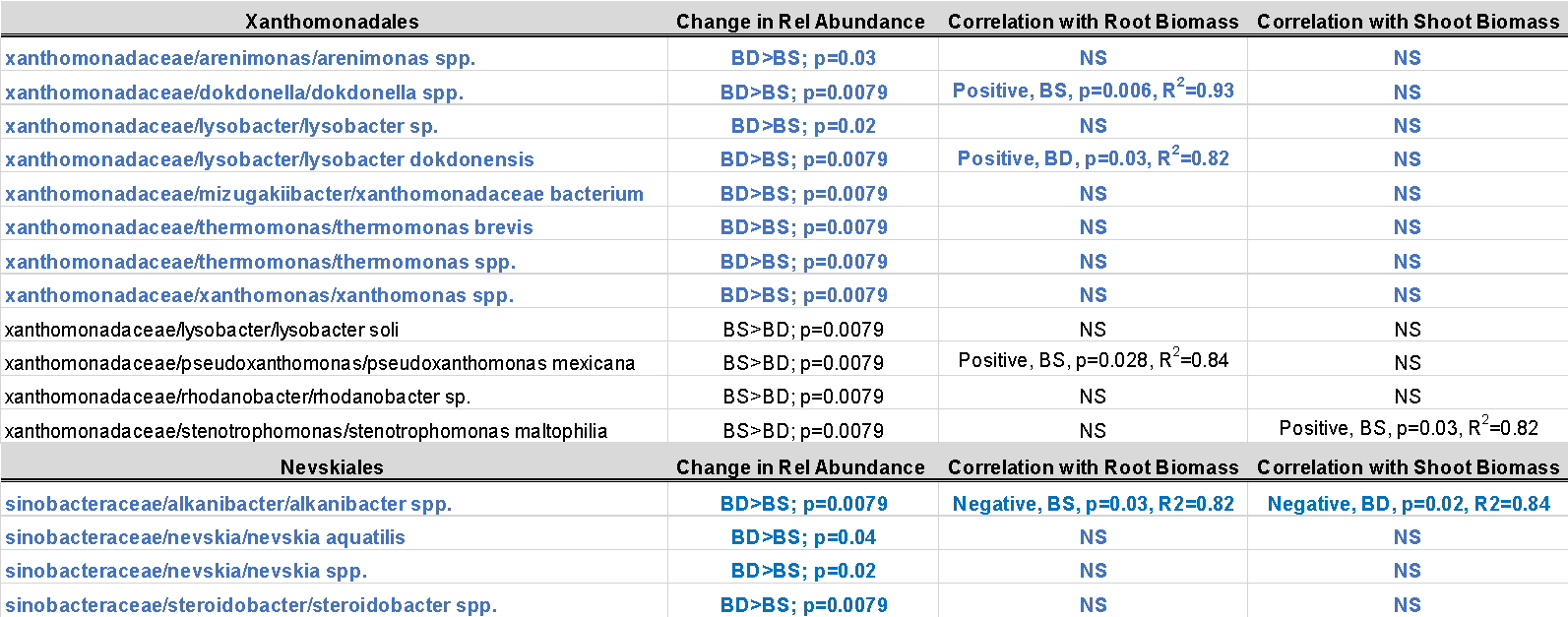
**Supplementary Table 10.** OTUs identified within the orders Xanthomonadales and Nevskiales that were significantly enriched (blue font) or reduced (black font) in BjSa + Dopamine (BD) relative to BjSa SM alone (BS). This data set was then screened for significant correlations with plant growth characteristics (root and shoot biomass). DNA used for the analysis was collected from the rhizosphere of apple seedlings planted into the respective treatments after 4 weeks.

**Figures**

**
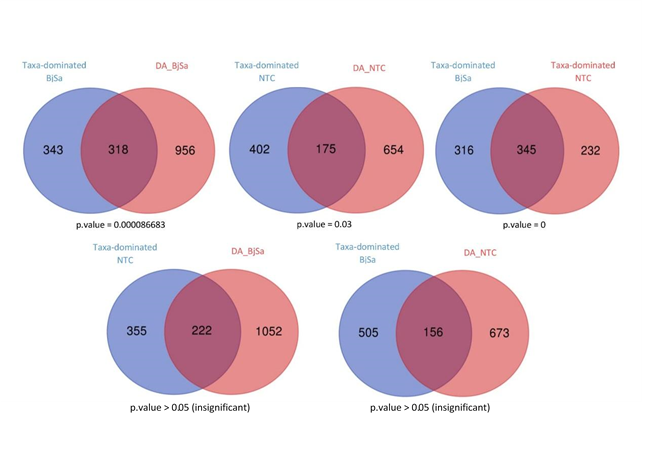
Supplementary Figure 1.** Venny diagram of the overlap between taxa-dominated enzymes (genus level) and differentially abundant (DA) enzymes in the treatment (Bjsa) and control (NTC) samples**.**

**Supplementary Figure 2.** Schematic illustration of the process of source metabolite selection and network reconstruction. The networks are comprised from metabolites denoted by circles that are connected by reactions (edges). A metabolite that is considered as an environmental resource is colored black. (A) Identification of source-metabolites in the meta-network, containing all cross-sample reactions. (B) Network reconstruction and prediction of environmental resources for two networks describing the root and soil environments. Each network contains all the differentially abundant enzymes in the corresponding environment. Common resources between a specific environments and the initial meta-network are denoted black with a colored rim. Green shades represent root environment, brown shades represent soil environments. Colored circles (without inner black "pupil") are source-metabolites identified only for the network of differentially abundant enzymes and not for the meta-network and are likely to represent biases formed from the gapped nature of a network which relies solely on differentially abundant enzymes. (C) The common environmental resources between the meta-network and each environment represents the predicted specific environment and were further used for network expansion. The algorithm starts with a set of source-metabolites acting as substrates – here the environmental proxy generated at stage B; it scans the reaction bank for feasible reactions for which all the possible substrates exist; all feasible reactions are added to the network, their products being the substrates for the next set of reactions. The network stops expanding when no feasible reactions are found. Thus, the full expansion of the network reflects both the reaction repertoire and the primary set of compounds (environmental proxy). Figure was taken from Tal et al, Microorganisms 2021, 9, 1838.
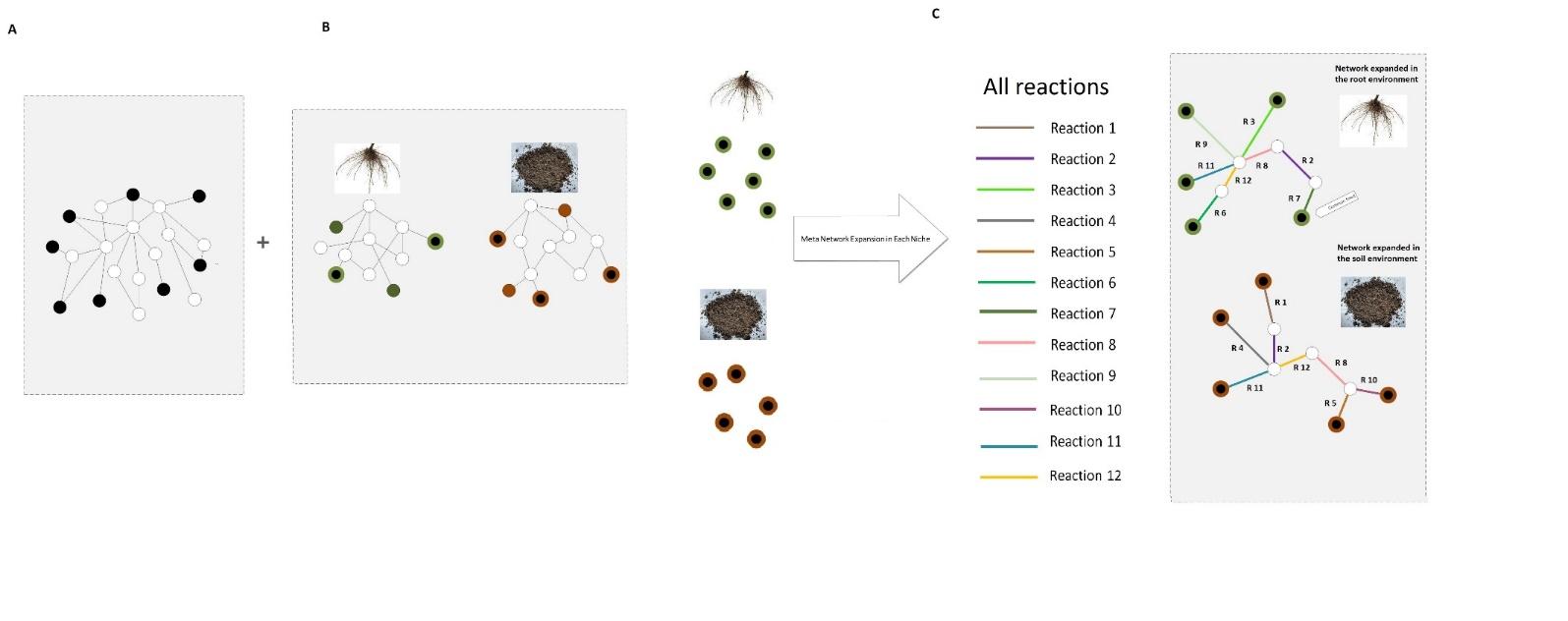


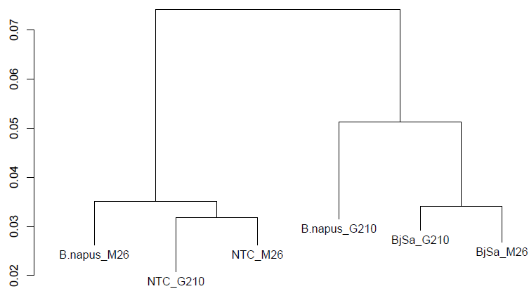

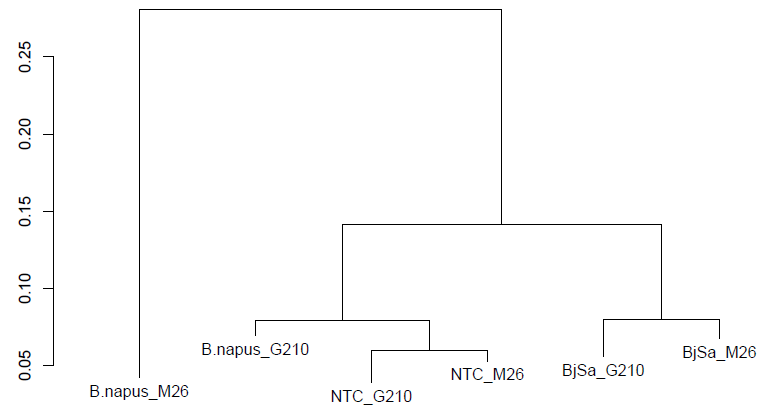


**Supplementary Figure 3.** Tree plots based on Bray Curtis distance matrices of treatments based on distribution of genera (left) and KO functions (right).

**
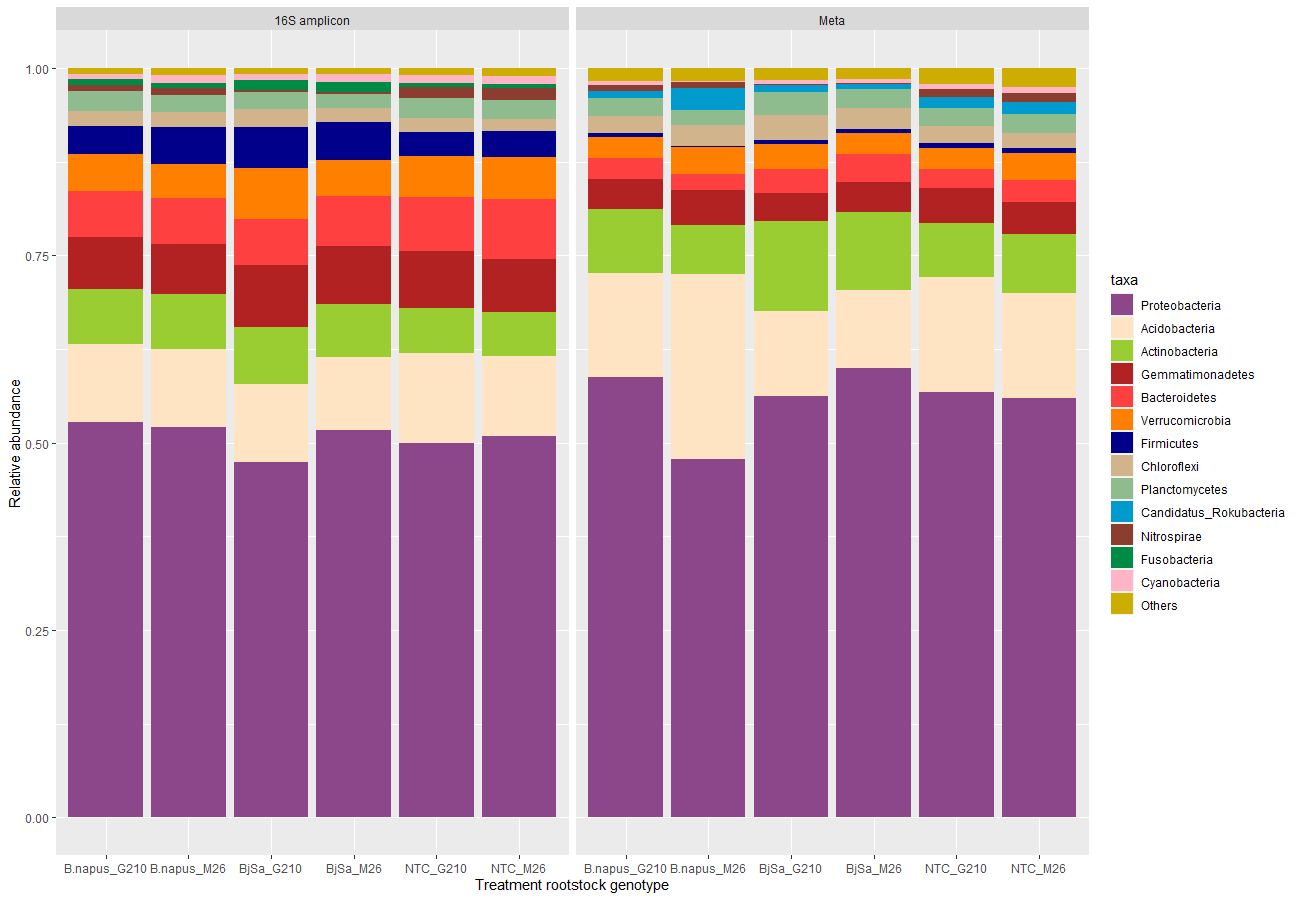
**

**Supplementary Figure 4.** Taxonomic distribution of bacterial phylum based on 16 rRNA amplicon sequences (left) vs shotgun sequencing (right). Amplicon data was taken from Somera et al 2021[^1^](#_heading=h.30j0zll).

**A. B.**


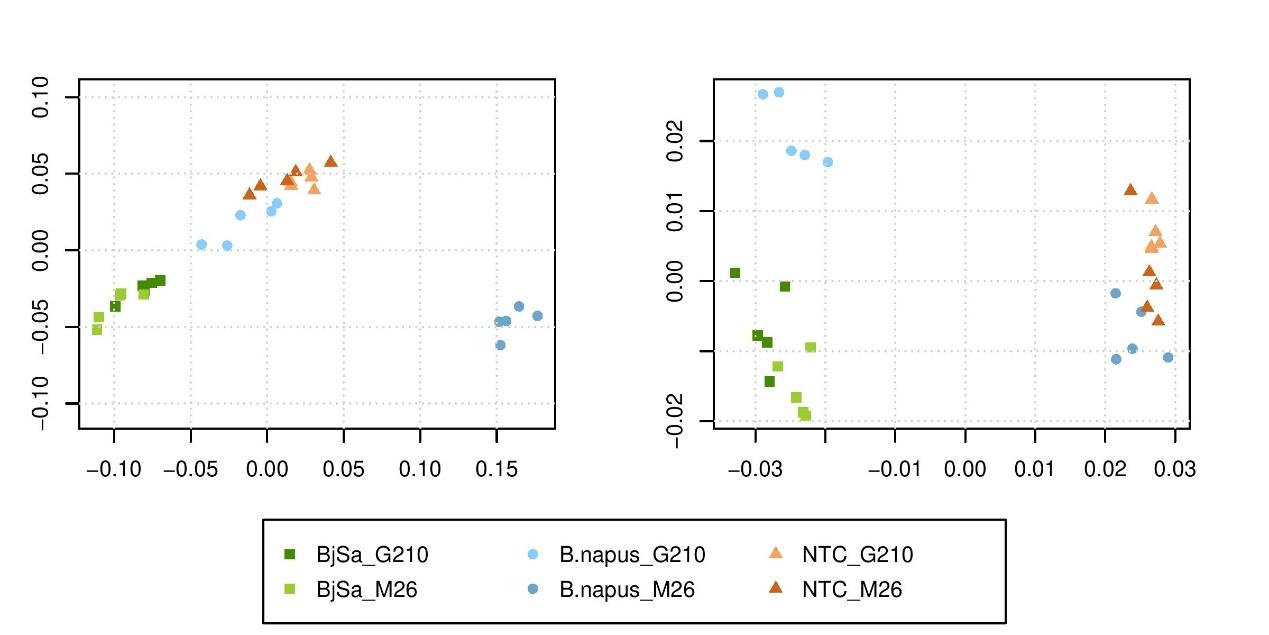


**Supplementary** **Figure 5.** Principal Coordinates Analysis (PCoA) plots of Bray-Curtis dissimilarities in the taxonomic (A) and functional (B) groups in root communities based on count tables derived from the metagenome analysis. A. Order level taxonomy. (B) Ordination pattern based on EC annotations. Both databases were filtered to at least 50 counts per feature, and rarefied.


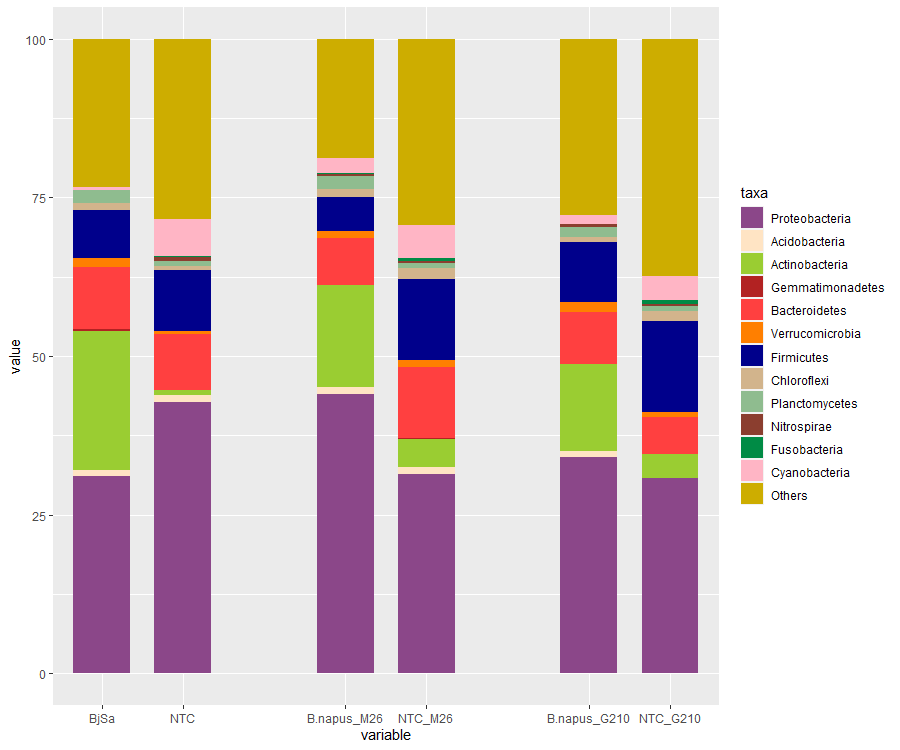


**Supplementary Figure 6.** Taxonomic (phylum level) distribution of treatment enriched genera. The full list of treatment enriched groups are provided in Supplementary Data 1.


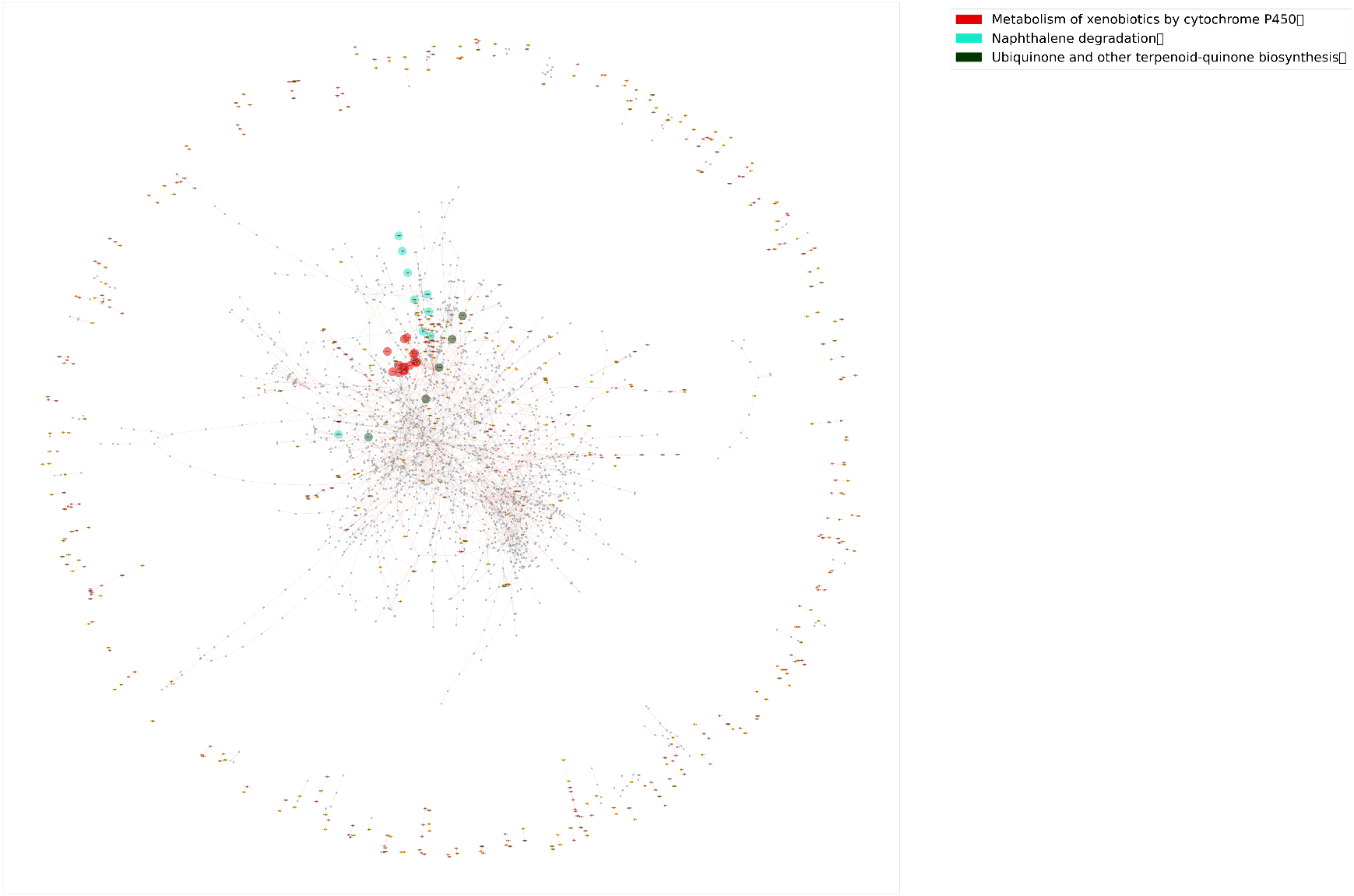


**Supplementary Figure 7.** Visualization of the networks representing the metabolic activity in NTC (control) versus BjSa SM-treated samples. Network was produced by simulating metabolic activities given a set of metabolic reactions (3060 enzymatic reactions) in control samples. Edges represent enzymes; nodes represent metabolites. Colored edges represent DA enzymes; light-colored nodes (green or orange) represent environmental sources unique to the sample type; dark-colored nodes represent network components unique to the sample type. Nodes' background colors (wider circles around the nodes) represent pathways that are enriched (FDR adjusted P value <= 0.05) with network components (nodes) that are unique to the treated samples.

**Supplementary Figure 8.** Catecholamine metabolism. Colored reactions are such that are dominated by Xanthomonadales in G.210 X BjSa samples and Nevskiales (genus *Solimonas*) in M.26 X BjSa samples. Enlarged compounds represent metabolites that are dependent on the respective groups. Pathway scheme was constructed according to the KEGG([9](#_heading=h.44sinio)) tyrosine metabolism pathway and illustrated using Escher tool([10](#_heading=h.2jxsxqh)).


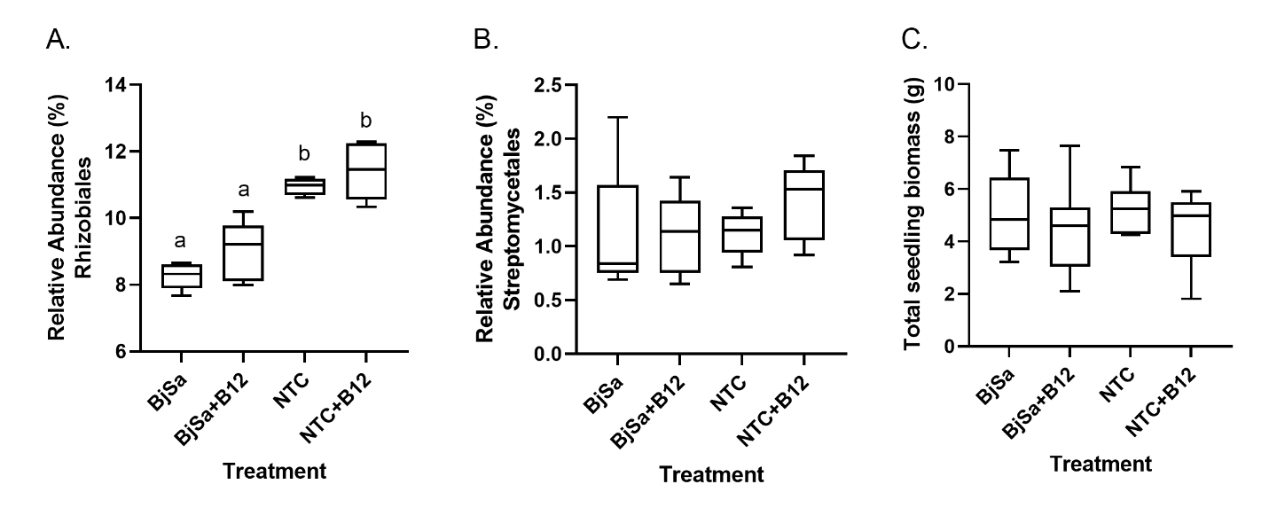


**Supplementary Figure 9.** **Box-and-whiskers plots** showing the relative abundance of all (A) Rhizobiales and (B) Streptomycetales, predicted by network analysis to be linked to the production of Vitamin B12 in SM-amended soil, and (C) seedling biomass measured upon harvest (8 weeks post planting). For A and B, 16S rRNA gene sequence data was obtained from DNA extracted from rhizosphere soil collected 4 weeks post-planting into soil amended with BjSa seed meal (SM), BjSa SM +Vitamin B12, an unamended control (NTC), or NTC + Vitamin B12 (n=5). In A, significance between treatments (as indicated by letter groups) was determined using One-way ANOVA test followed by Tukey’s multiple comparisons test: p < 0.0001 for BjSa vs. NTC and NTC + B12; p = 0.001 for BjSa + B12 vs NTC; p = 0.0001 for BjSa + B12 vs NTC+ B12. Values plotted represent the 25th percentile, the median, the 75th percentile; whiskers extend to the minimum and maximum.

**A. B.**
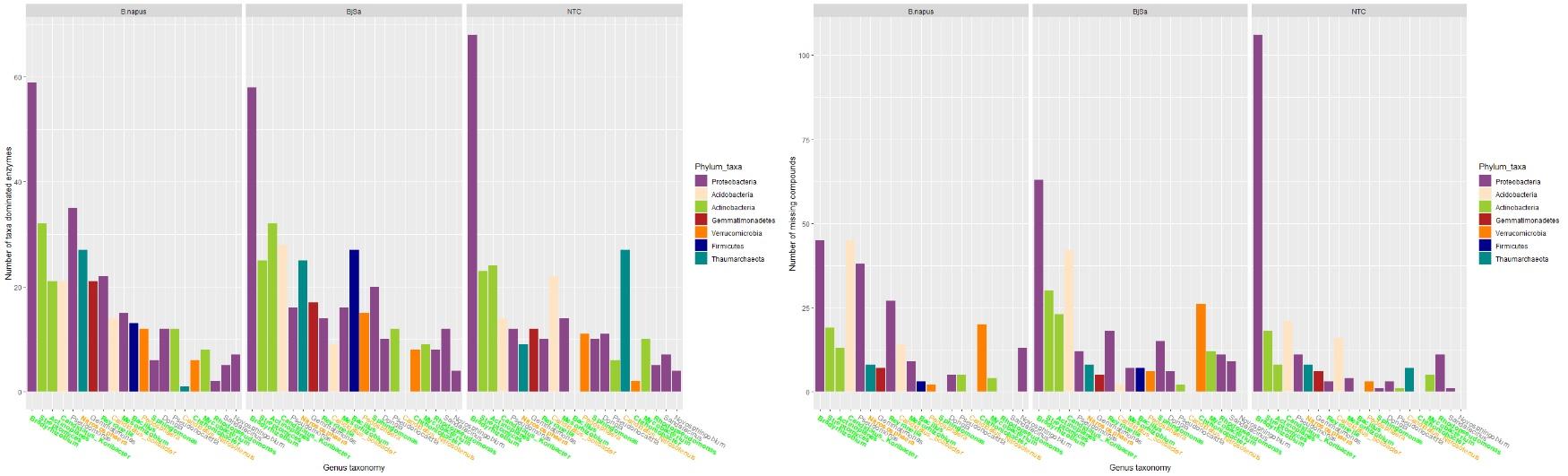


**C.**
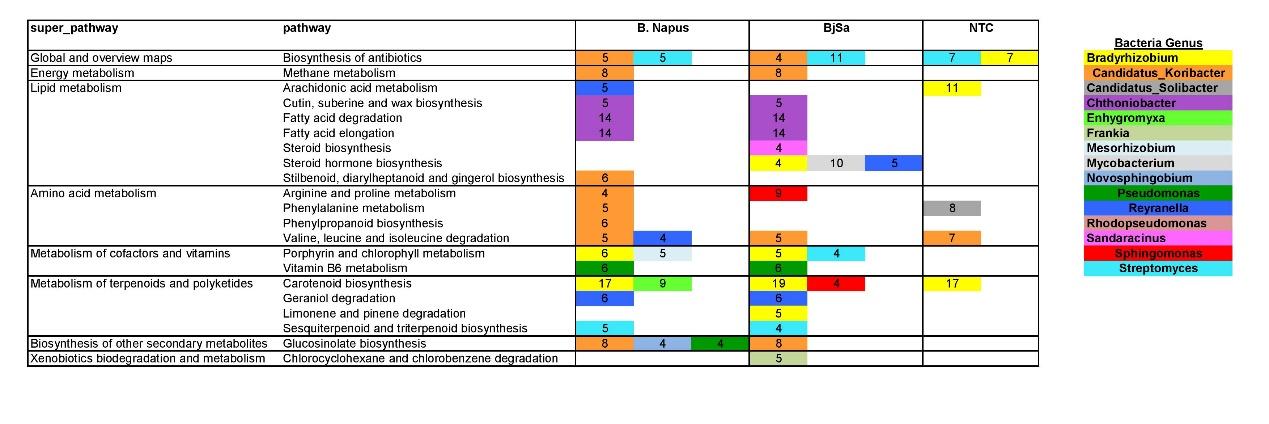


**D.**

**
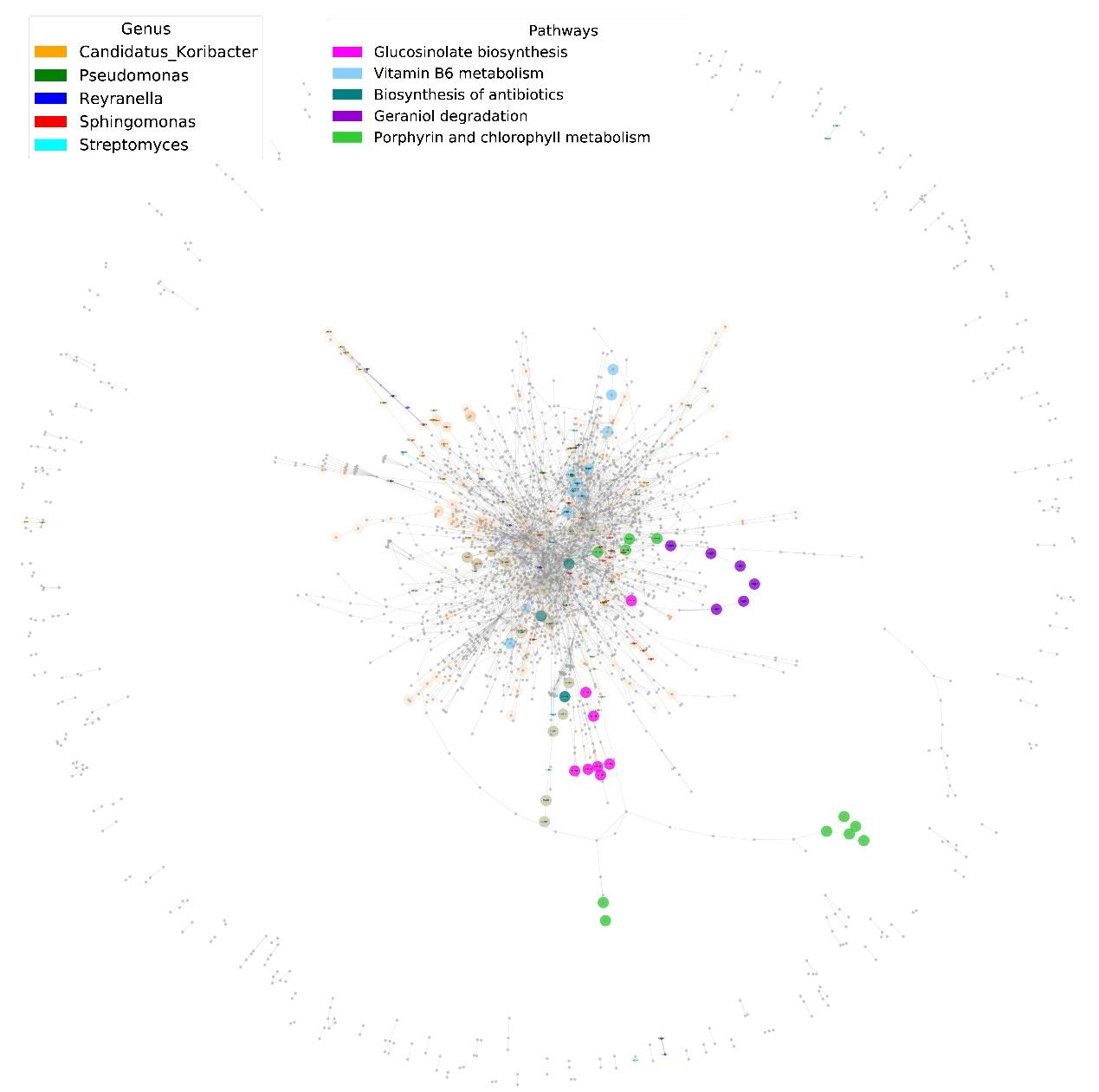
**

**Supplementary Figure 10.** Distribution of genus-level taxa-associated functions as determined for the different treatments (G210 rootstocks).A. Enzymatic functions that are dominated by specific taxonomic groups, B.Taxa-dependent compounds and C. Pathway distribution of taxa-dependent compounds**.** Colored labels of taxonomic groups indicate significant abundance in the SM treated (green) or control (orange) samples. Pathways with at least four taxa-dependent compounds are detailed. D. Taxonomic stratification of microbial functions. Visualization of genus-level dominated functions (enzymes & compounds) in a network representing the overall metabolic activity in BjSa samples treated soil. Colored edges and nodes in the network represent taxa-dominated enzymes and taxa-dependent compounds in BjSa X G210, respectively. Compounds associated with the pathways in the table on top, are indicated by nodes' background color.

**References**

1. Somera TS, Freilich S, Mazzola M. Comprehensive analysis of the apple rhizobiome as influenced by different Brassica seed meals and rootstocks in the same soil/plant system. *Applied Soil Ecology* **157**, 103766 (2021).

2. Kinney KS, Austin CE, Morton DS, Sonnenfeld G. Norepinephrine as a growth stimulating factor in bacteria--mechanistic studies. *Life Sci* **67**, 3075-3085 (2000).

3. Simon M*, et al.* Phylogenomics of Rhodobacteraceae reveals evolutionary adaptation to marine and non-marine habitats. *The ISME journal* **11**, 1483-1499 (2017).

4. Wang W*, et al.* Consistent responses of the microbial community structure to organic farming along the middle and lower reaches of the Yangtze River. *Scientific reports* **6**, 35046 (2016).

5. Asker D, Beppu T, Ueda K. Sphingomonas jaspsi sp. nov., a novel carotenoid-producing bacterium isolated from Misasa, Tottori, Japan. *Int J Syst Evol Microbiol* **57**, 1435-1441 (2007).

6. Wei J*, et al.* Seasonal Dynamics and Starvation Impact on the Gut Microbiome of Urochordate Ascidian Halocynthia roretzi. *Research Square*, (2020).

7. Hausmann B*, et al.* Peatland Acidobacteria with a dissimilatory sulfur metabolism. *The ISME journal* **12**, 1729-1742 (2018).

8. Wang H*, et al.* Bacterial, archaeal, and fungal community responses to acid mine drainage-laden pollution in a rice paddy soil ecosystem. *The Science of the total environment* **616-617**, 107-116 (2018).

9. Kanehisa M*, et al.* KEGG for linking genomes to life and the environment. *Nucleic Acids Res* **36**, D480-484 (2008).

10. King ZA, Dräger A, Ebrahim A, Sonnenschein N, Lewis NE, Palsson BO. Escher: A Web Application for Building, Sharing, and Embedding Data-Rich Visualizations of Biological Pathways. *PLoS computational biology* **11**, e1004321 (2015).
